# Integrating family planning with nutrition and other sexual and reproductive health services in low-income and middle-income countries: findings from a scoping review

**DOI:** 10.1136/bmjgh-2024-017482

**Published:** 2025-05-30

**Authors:** Sachin Shinde, Cara Yelverton, Nazia Binte Ali, Uttara Partap, Moussa Ouédraogo, Innocent Yusufu, Iqbal Shah, Wafaie Fawzi

**Affiliations:** 1 Department of Global Health and Population, Harvard University T H Chan School of Public Health, Boston, Massachusetts, USA; 2 Maternal and Child Health Division, International Centre for Diarrhoeal Disease Research, Dhaka, Bangladesh; 3 University of California San Francisco, San Francisco, California, USA; 4 Africa Academy for Public Health, Dar es Salaam, Tanzania

**Keywords:** Global Health, Health services research, Nutrition, Review

## Abstract

**Introduction:**

Integrating family planning with nutrition could address unmet family planning needs and malnutrition in low-income and middle-income countries (LMICs). Integrating family planning with sexual reproductive health (SRH) services may provide valuable insights as well. This scoping review synthesised evidence from published and grey literature on interventions integrating family planning with nutrition or SRH services in LMICs.

**Methods:**

We searched MEDLINE, EBSCO and Google Scholar for studies on integrated family planning, nutrition or SRH services, including codelivery or referral-based models. Evidence was analysed using narrative synthesis.

**Results:**

We reviewed 109 articles; most family planning within broader health initiatives, particularly targeting maternal and child health and health system strengthening efforts. Integration strategies varied, generally involving codelivery of services or referral systems linking different service platforms and using diverse health providers. Outcomes were often reported by individual components, concentrating on specific services like family planning use and less so on nutrition or overall maternal and child health, thus limiting a complete understanding of the integrated intervention’s impact. Qualitative and process evaluations primarily explored the acceptability and feasibility of integration, highlighting challenges in service delivery, stakeholder engagement and training and supervision gaps.

**Discussion:**

Our findings suggest three evidence-based priorities for improving the integration of family planning, nutrition and SRH services: (1) standardising definitions and frameworks for integration typologies; (2) investing in workforce training and (3) strengthening supply chains. These align with implementation challenges recurrently observed across studies. The review also underscores the necessity for more rigorous research focused on the effectiveness and cost-effectiveness of integrated approaches, providing critical insights for decision-makers aiming to optimise health service delivery in resource-limited settings.

**WHAT IS ALREADY KNOWN ON THIS TOPIC**

- Evidence on the impact of integrating family planning with nutrition and/or sexual reproductive health (SRH) programmes remains limited.

**WHAT THIS STUDY ADDS**

- We found that family planning and nutrition interventions are often integrated into broader health initiatives, such as maternal and child health or health system strengthening efforts, using diverse platforms like community, facility, school and digital services.
- The review highlights a significant gap in integrating family planning with nutrition or SRH services due to insufficient planning and evaluation, and lack of human resources, infrastructure, financing, underscoring the need for well-structured initiatives with built-in evaluation to ensure efficiency, sustainability and impact.
- While family planning and SRH integration, especially for HIV, is well-established, this review highlights: (1) significant gaps in non-HIV SRH integration, (2) opportunities to apply family planning and SRH lessons to family planning and nutrition (eg, using community health workers) and (3) emerging challenges in sustaining integration due to health system pressures (eg, workforce shortages).

**HOW THIS STUDY MIGHT AFFECT RESEARCH, PRACTICE OR POLICY**

- The review identifies key gaps in knowledge and future research opportunities, including the impact of various integration strategies on service coverage, efficiency, costs and overall health outcomes.

## Introduction

 Unmet contraceptive needs, malnutrition among women of reproductive age and inadequate sexual and reproductive health (SRH) care persist globally. In 2019, around 218 million women (15–49 years old) in low-income and middle-income countries (LMICs) had unmet contraceptive needs—43% adolescent girls and 24% women aged 20–45 years, with significant access and coverage disparities.^1 2^ Additionally, an estimated 133 million women in LMICs require but do not receive services for SRH issues.^2 3^ Concurrently, LMICs confront severe malnutrition challenges, affecting individuals and communities across their lifespan. The prevalence of underweight, overweight and obesity among women of reproductive age stands at 15.2%, 19.0% and 9.1%, respectively,^4^ with anaemia affecting half a billion women globally.^5^,^7^

Considering the significant biological, economic and health system benefits, integrating family planning (FP) and nutrition services is crucial for effectively addressing the challenges of unmet FP needs and suboptimal nutrition.^8^,^12^ Contraceptive use optimises birth spacing, reducing maternal anaemia and child stunting, while shared service platforms like antenatal care improve efficiency. Adequate maternal nutrition lowers the risks of anaemia, preterm birth and low birth weight, positively influencing contraceptive use and adherence. Economically, better nutrition reduces healthcare costs associated with maternal and neonatal complications, enhancing the cost-effectiveness of integrated services.^13^ Leveraging antenatal and postnatal care for both nutrition and FP interventions strengthens health systems and advances Sustainable Developmental Goals (SDGs) 2 and 3.

Furthermore, such an integrated approach can significantly reduce maternal mortality and morbidity, enhance child development and break the intergenerational cycle of malnutrition.^14^ Moreover, combining FP and nutrition interventions can empower women by enhancing their educational opportunities, workforce participation and addressing malnutrition through improved economic productivity and food security. This, in turn, supports the achievement of both international and national development objectives.^9^ Previous efforts of integrating FP with other health services suggest that health systems can be strengthened by streamlining service delivery, reducing duplication and improving overall programme efficiency, thereby reducing costs.^11^ Lessons from such integration efforts could also apply to combining FP with nutrition programmes.

Despite the growing interest in integrated FP and nutrition interventions,^8^ there remains a significant gap in comprehensive understanding of their scope and nature.^12 14^ The debate on the value and scope of integration between FP and nutrition centres on whether integration should: (1) combine these two distinct services at the point of care, potentially overloading providers; (2) link services via referrals, which could result in dropouts or (3) prioritise one domain over the other. Furthermore, previous efforts combining FP with SRH services could provide important lessons for the integration of FP and nutrition interventions. While systematic reviews have examined FP integration with maternal and child health, HIV and other health services,^10 11 15 16^ evidence on integrating FP and nutrition services remains limited. These reviews often omit broader SRH services (eg, abortion, STI screening and care), creating a gap in understanding how FP and broader SRH services could be integrated with broader maternal and child health programmes. This is particularly important given that the WHO considers FP a key component of SRH. Moreover, the overlap between abortion and FP services is more pronounced in the context of postabortion FP than in the provision of abortion itself, further underscoring the need for a more integrated approach.

The only identified synthesis in this area focused on grey literature from United States Agency for International Development (USAID)-funded programmes integrating FP with nutrition and food security, excluding other key non-HIV SRH services.^15^ As understanding of these integrated approaches evolves,^10^,^1214^ synthesising existing evidence is critical to determine effective practices and future research opportunities, ensuring that investments are channelled towards the most effective strategies.

Therefore, this scoping review aims to synthesise evidence from both published and grey literature on integrated interventions of FP services with nutrition interventions and/or other SRH services, targeting adolescents and women of reproductive age in LMICs since 2000.

## Methods

**Figure 1:**
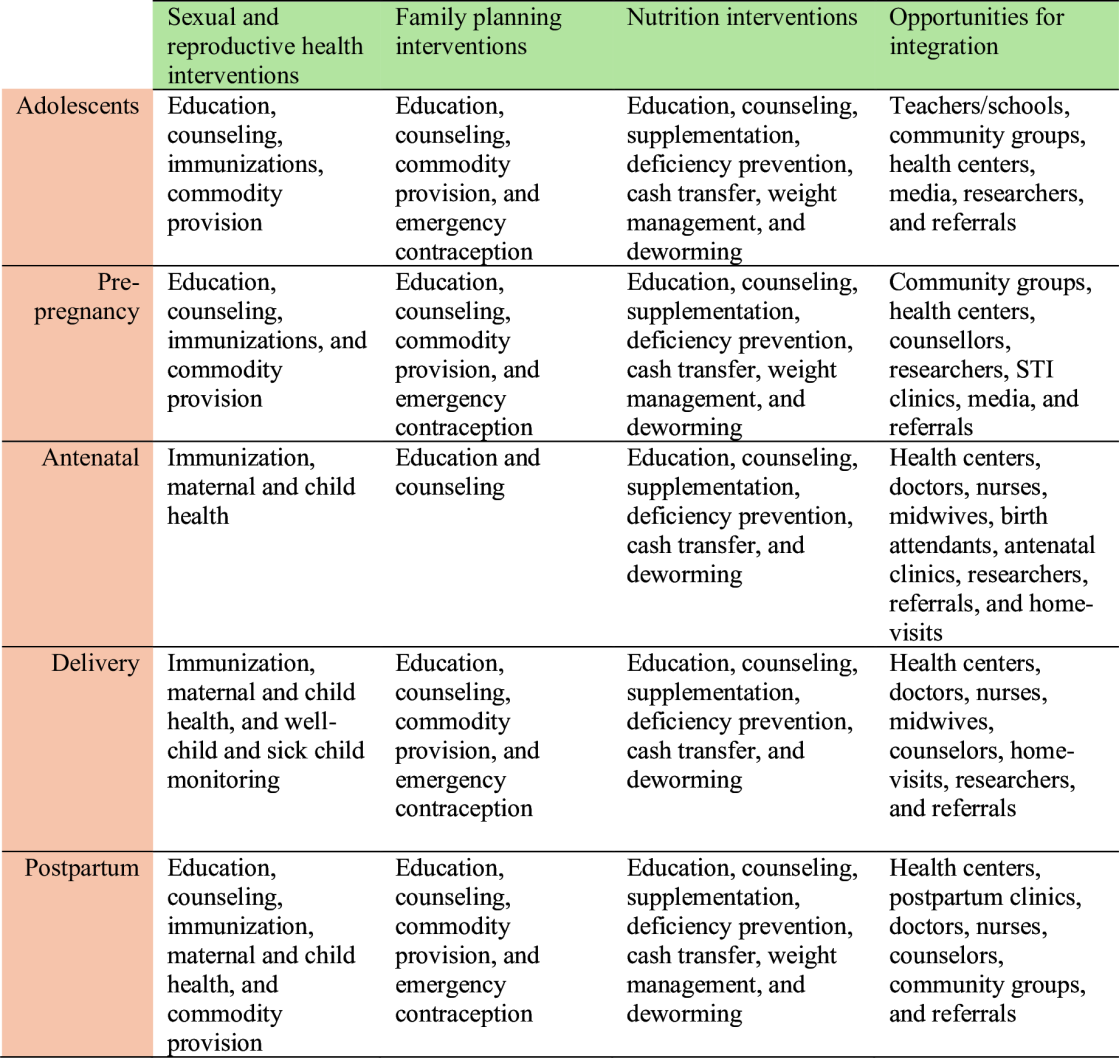
Framework for evidence synthesis of integrated family planning and nutrition services. STI, Sexually Transmitted Infections.

We prospectively registered the protocol (Open Science Framework, DOI:10.17605/OSF.IO/KSPT4). The review is guided by a conceptual framework (figure 1), covering integration platforms, providers and target populations. This framework defines integrated programmes where FP and nutrition or SRH services are provided either (a) by the same provider, (b) at the same contact/entry point or (c) through the same service delivery platform. Additionally, the framework acknowledges integrated interventions across the life-course, from adolescence to adulthood and various reproductive phases from non-pregnant to postpartum.

### Eligibility criteria

This review included studies both published or unpublished in English, conducted in LMICs and meeting the following criteria: randomised controlled trials (RCTs), controlled before-and-after studies, evaluations with qualitative, mixed-methods or case study designs, or implementation research approaches with or without control or comparison group; integrating FP services with nutrition and/or other SRH programming; involving reproductive age women; and addressing at least one outcome on either or both of the domains being integrated.

As outlined in figure 1, FP services include a range of interventions designed to help individuals and couples space pregnancies, such as counselling, contraceptive methods (eg, oral contraceptives, intrauterine devices, condoms), and fertility awareness education. Nutrition services for women include preconceptional, prenatal and postnatal nutrition education, counselling, micronutrient supplementation (eg, iron, folic acid), dietary advice to improve maternal health, weight management support, and interventions to address malnutrition, anaemia and other nutrition-related health issues during adolescence, pregnancy and post partum. SRH services include maternal immunisation, contraception, preconception care, antenatal and postnatal care, safe abortion services, sexually transmitted infection prevention and treatment, services related to SRH and comprehensive sexuality education and counselling.^17^ As FP is a core component of SRH services, overlap between FP and abortion services is more in postabortion FP than in provision of abortion. To address the possible overlap, our scoping review included both FP-specific and broader SRH interventions where relevant and explicitly described. We categorise and discuss these intersections to highlight opportunities for more integrated and comprehensive service delivery.

We included both FP–nutrition and FP –SRH integration because: (1) they share target populations (eg, postpartum women), (2) often use identical delivery platforms (eg, child health visits), and (3) address overlapping health determinants (eg, poverty). Although aspects of FP and SRH integration have earlier been studied, further evidence on broader and more comprehensive dimensions of this integration could be essential for informing approaches to FP and nutrition integration.

This review also included grey literature such as unpublished research and evaluation reports to ensure comprehensive understanding of available evidence. The scoping review included studies published since 2000 to ensure relevance to contemporary health systems and integration approaches. This period aligns with global milestones such as the SDGs, reflecting shifts in FP, nutrition and SRH priorities. It also captures improved data quality, standardised methods and technological and sociocultural changes, enhancing the applicability of findings to current and future efforts in LMICs.

Studies exclusively focusing on the integration of FP with HIV services were excluded due to existing extensive literature.^16^ Studies addressing the integration of FP with food insecurity programming were excluded to focus on direct nutrition services, ensuring a clearer synthesis of FP and nutrition integration without the influence of broader structural interventions like cash transfers, food aid and agricultural support. Observational studies were excluded, including cohort, case–control and cross-sectional designs, as well as editorials, commentaries, opinions and review articles. While observational studies could have provided valuable insights into baseline conditions and long-term trends, their exclusion was necessary to maintain the focus on intervention-specific outcomes and the practical aspects of integration.

### Databases, search strategy and sources of articles

A comprehensive approach was employed to identify potentially relevant studies. MEDLINE (through PubMed), Global Health (through EBSCO) and Google Scholar were comprehensively searched. The search strategy used key terms related to the target population, type of intervention, study design, and settings. Search terms were tailored for each database, with the PubMed search strategy detailed in online supplemental file 1.

In addition to the electronic database search, bibliographies of retrieved studies and pertinent systematic reviews were manually checked to identify other relevant studies. Furthermore, supplementary searches encompassed online resources like ClinicalTrials.gov and organisational websites (WHO library, UNICEF and Knowledge SUCCESS). Records retrieved from electronic databases and other searches were imported into Covidence (Veritas Innovation, Melbourne, Australia). Titles and abstracts and the full texts were screened by two independent researchers. Any disagreements about eligibility were settled through discussion. Study exclusions were documented and summarised using the Preferred Reporting Items for Systematic Reviews and Meta-Analyses flow diagram for scoping reviews.^18^

### Data extraction

We designed and piloted the data extraction template on five studies to refine it further. The template included details on study design, sample size, intervention description, service provider details, comparator/control if applicable, outcomes assessed, main results, process evaluation findings (eg, coverage, uptake and quality of services), facilitators and barriers to integration, and considerations and measures of sustainability. Two authors (CY and NBA) independently extracted data using the finalised template, with a third reviewer (SS) verifying 50% of entries for consistency. Inter-rater reliability was assessed through dual extraction of a subset of studies, with disagreements resolved by discussion.

### Synthesis of evidence

We systematically synthesised findings from all included studies, in the text and table, using the Synthesis Without Meta-analysis guidelines.^19^ Our review focused on examining approaches to integration and summarising methodological trends, rather than synthesising effect estimates through meta-analysis. Meta-analysis was not feasible due to heterogeneity in intervention, inconsistent effect measures (eg, percentages vs ORs) and methodological diversity (eg, RCTs vs process evaluations). Instead, we categorised results by integration type (eg, FP-maternal health) and used thematic analysis to identify cross-cutting lessons. Intervention effects were summarised using mean differences for continuous variables and percentage and/or percentage points differences for binary outcomes. We presented a summary count detailing the direction of effects for each outcome. Qualitative studies were analysed using thematic analysis and narrative descriptions of each theme are presented.

Using Let Evidence Guide Every New Decision guideline, we systematically evaluated research evidence for relevance, reliability, validity and applicability, considering different sources like quantitative and qualitative studies.^20 21^ Each included study was assessed across four components—relevance, reliability, validity and applicability—rated as ‘low’, ‘average’ or ‘high’. Studies were classified as ‘strong’ in quality if all components were rated ‘high’, ‘moderate’ if one component was rated ‘low’ and ‘weak’ if two or more components were rated ‘low’. Incorporating quality appraisal in scoping reviews is not typical, but it was essential in this case to ensure a robust synthesis of diverse study designs, given the complexity of combining RCTs, quasi-studies, and qualitative and process evaluations.

We the authors—SS, CY, NBA, UP, MO, IY, IS and WF—represent a diverse team of global health researchers and practitioners with expertise in implementation science, public health, nutrition and SRH. Our team includes academic researchers, programme implementers and policy advisors, with the majority based in or primarily working in LMICs. This composition ensured contextual relevance and grounded perspectives in LMIC health systems. Our collaboration was driven by shared commitments to interdisciplinary integration, equity and evidence-based policy. Detailed reflections on our positions, biases and roles in shaping the review are provided in the online supplemental file 2.

#### Patient and public involvement

No members of the public were involved in this research.

## Results

### Description of studies

**Figure 2:**
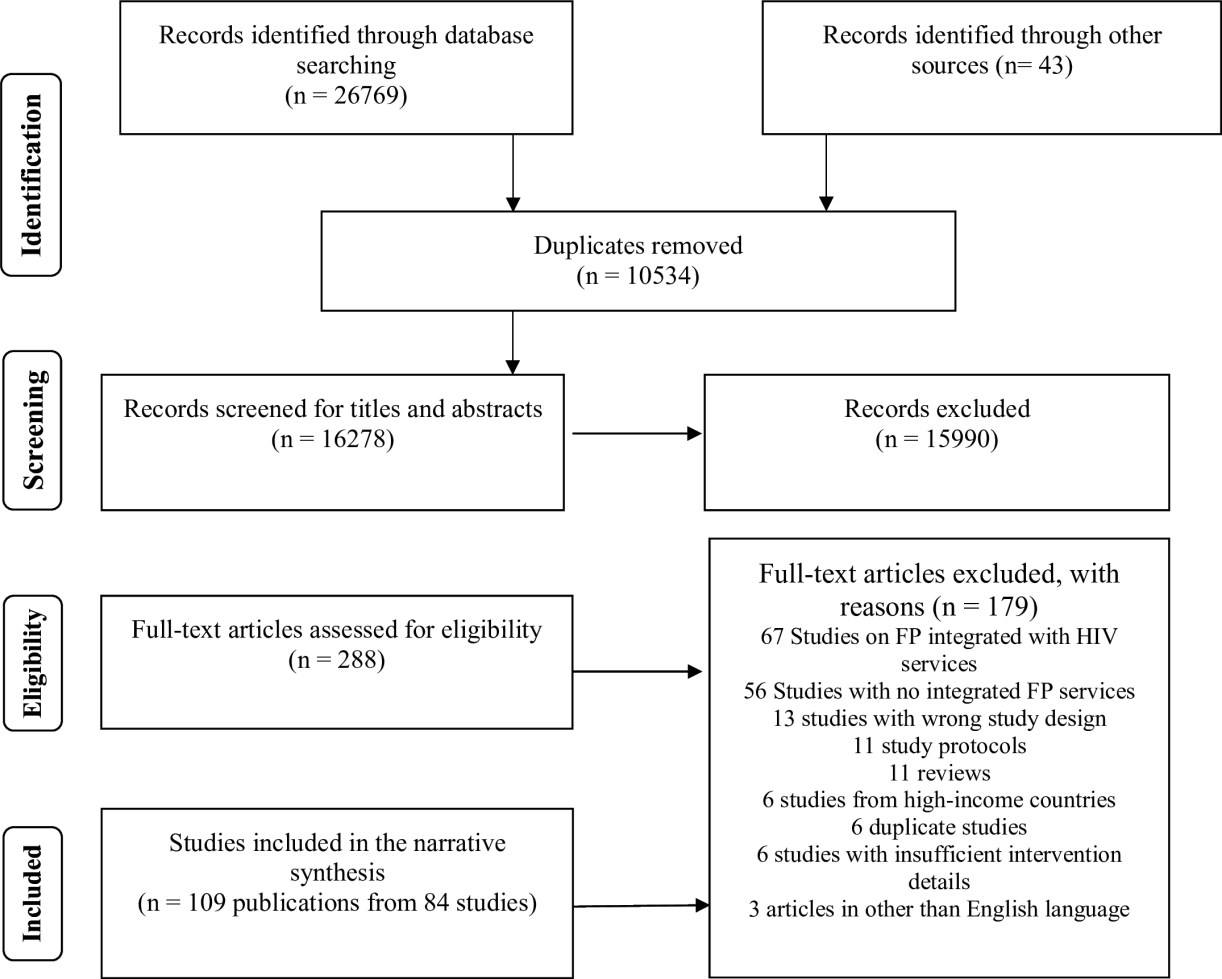
Flow chart for the literature search for integrated family planning (FP) and nutrition services.

Our search yielded 16 278 unique records (figure 2), with duplicates removed using Covidence. After title and abstract screening, 15 990 records were excluded for not meeting the eligibility criteria, and 288 full text articles were reviewed. For studies with multiple publications, we retained the most comprehensive one (eg, largest sample size, longest follow-up) or those reporting unique outcomes. Initial decisions were made by two reviewers (SS and CY), with conflicts resolved by senior authors (WF and IS). Ultimately, 109 articles describing 84 distinct integration efforts of FP with nutrition or SRH services met inclusion criteria. Key study characteristics are summarised in online supplemental file 3.

### Study design, setting and population

Of the 109 articles,^22^,^130^ 17 were based on clustered RCTs, 16 quasi-experimental studies, 14 qualitative studies and 3 individually RCTs (online supplemental file 3). The remaining 59 articles were based on programme evaluations using preintervention and post-intervention or only postintervention designs and process data.

The 84 studies were from 42 countries across WHO regions, with 55 conducted in the African region, 15 in the South-East Asia region, 10 in the Eastern Mediterranean Region, three in the Region of the Americas and 1 in the European Region. Overall, 24 were aimed at all women of reproductive age, 21 were specifically targeted at postpartum women, 17 were focused on pregnant women, 11 covered both pregnant and postpartum women, 6 involved only women seeking abortion care and 5 were conducted with only adolescents (aged 12–19 years).

### Theoretical framework for the interventions

Of the 84 integration efforts, 9 explicitly used a theoretical framework to inform the integration. Two studies referred to the health beliefs model^59 62^; while one study each cited the transtheoretical model of behavioural change^47^ and the theory of planned behavior.^74^ One study used the USAID’s maternal and child health integrated programme as the theoretical underpinning for integrating FP and maternal and child health services,^67^ while one study each designed its intervention based on UNICEF and WHO’s Baby Friendly Initiative,^86^ WHO’s antenatal care model^93^ or the recommendations from the International Conference of Population and Development 1994.^110^ One study from India referred to the National Rural Health Mission Framework to integrated FP and maternal and child health services.^63^ Remaining studies reported the demand-side unmet needs as the motivating factor for integrating FP services with maternal and child health and other health services.

### Intervention focus

Out of 84 integration efforts, 70 integrated FP into maternal and child health programming (online supplemental file 3). Among these, 41 studies integrated FP into overall maternal and child health services, 13 into child immunisation and care, 8 into postpartum services and 7 into antenatal services. One study integrated FP into broader reproductive health services. Among the remaining 14 studies, 7 integrated FP into postabortion care, 5 integrated FP education into comprehensive sexuality education for adolescents and youth and 2 included FP as components of health systems strengthening efforts. Interventions focusing solely on FP and nutrition integration were uncommon and typically embedded within broader service packages.

#### Integration of FP and packages of maternal, neonatal and child health

43 studies described integration of FP services into maternal and child health programming to improve birth intervals for better maternal and child health outcomes. Among these, 16 included nutrition services for mothers and infants, 7 focused on child nutrition and 20 did not include any nutrition components. The maternal and child health packages included: (1) awareness and care for non-pregnant women, (2) pregnancy care (immunisation, nutrition education and/or counselling, micronutrient supplementation, management and prevention of maternal infections and detection of maternal complications), (3) labour and newborn care (breastfeeding counselling), (4) postpartum care (maternal diet and nutrition and immunisation), and (5) child health (growth monitoring, immunisation, vitamin A supplementation, deworming, and prevention and management of childhood illnesses). Integrated FP services emphasised birth spacing through education, counselling and contraceptive distribution, complemented by group discussions, multimedia campaigns, and social marketing to promote short-acting modern contraceptives and link women to clinic-based care.

The integration of services varied by modality—ranging from same-day, colocated service delivery to referral-based approaches—based on factors such as resource availability, infrastructure, national policies, facility types and workforce availability. Among the 43 interventions, 17 were conducted in community settings, 11 combined facility and community delivery, 6 were provided at clients’ homes and health facilities and 5 were exclusively at clients’ homes. Four community-based interventions included referrals to nearby health facilities. Front-line health workers such as community nurses, community health workers, midwives, health extension workers and volunteers primarily implemented community-based activities, focusing on education, counselling and limited contraceptive provision (eg, condoms and pills). Clinic-based FP services were administered by doctors, nurses, counsellors and midwives, emphasising counselling and long-acting reversible contraceptives and complication management.

Various efforts in Bangladesh, Ethiopia, India and Nepal aimed to integrate FP into existing maternal and child health programming. This included colocating FP with maternal and child health services, training healthcare workers, especially front-line health workers, to provide both FP counselling and limited contraceptives and adopting client-centred approaches empowering women with FP choices alongside essential healthcare services for themselves and their children.

#### Integration of antenatal care and FP

Seven integration efforts of FP into antenatal care capitalised on regular client–provider interactions during pregnancy,^31 39 44 65 85 93 95^ aiming to facilitate postpartum FP initiation, with three studies describing nutrition services.^31 39 44^ Antenatal visits, typically ranging from 2 to 6 visits, encompassed health assessment (eg, anthropometric measurements, haemoglobin levels assessment and blood pressure monitoring), education on prevention and management of sexually transmitted infections, detection and management of pregnancy complications, health promotion and disease prevention (including vaccines, malaria prevention and treatment, nutrition counselling, and iron and folic acid supplementation), and birth preparedness. FP components focused on education and counselling by clinic-based nurses, midwives or community health workers, employing group and individual sessions with aids like storytelling and brochures. A few studies also included counselling on postpartum implant insertion for interested clients during antenatal visits, followed by follow-up appointments. In a quasi-experimental study in Guinea,^44^ enhanced antenatal counselling included a single face-to-face session on postpartum FP, lasting 15–20 min, alongside routine counselling covering maternal nutrition, childbirth preparedness and immunisation. Conducted by trained providers in the antenatal care unit, this session used contraceptive samples and a toolbox for guidance.

#### Integration of postpartum care and FP

Among eight studies of postpartum FP services,^27 29 42 49 56 73 80 105^ one described a nutrition component.^27^ Most studies cited the motivation for integrating FP into postpartum care as reducing adverse health outcomes for mothers and children, and meeting unmet FP needs and streamlining care. Postpartum visits covered infant care, breastfeeding guidance, fertility after lactation, psychological well-being, physical recovery, chronic disease management, child immunisation and nutrition education and/or counselling to mothers. Up to four visits occurred in the early postpartum period (first 6 weeks), followed by up to three visits over the subsequent 18 months.

The postpartum FP services included workshops for medical professionals, such as doctors, nurses, midwives and hospital staff, covering FP counselling and long-acting reversible contraceptive provision. Additionally, informational materials and videos were displayed in waiting areas, and counselling sessions were offered to women and their partners, including group, couple and one-on-one sessions. Contraceptives of choice were provided to women along with follow-up care and referrals. In a multisite study across six countries, FIGO (International Federation of Gynecology and Obstetrics) trained healthcare providers in public hospitals to offer postpartum intrauterine contraceptive devices via cascade trainings. FIGO intervention was evaluated in three countries, including Nepal,^106^,^109^ Sri Lanka^84 126^ and Tanzania.^67 102 114 115^ The intervention included providing FP counselling and informational materials during antenatal care visits and providing postpartum intrauterine device following delivery to women who requested for it. In another randomised trial in Guatemala, nurses provided free contraceptives (condoms, pills, medroxyprogesterone syringes, implants), follow-up care and referrals at postpartum home visits alongside routine care.^72 73^

Two studies specifically focused on integrating Lactational Amenorrhea Method into postpartum services, employing strategies like training healthcare workers, providing scheduled home visits for breastfeeding education and support and using multimedia promotion including television and radio spots, brochures, and flipcharts.^42 46^

#### Integration of child healthcare and immunisation and FP

13 interventions integrated child healthcare, immunisation and FP to address mothers’ FP needs child health visits; only two community-based interventions included vitamin A supplementation for children. Clinic-based services focused on immunisation and growth monitoring, while community initiatives emphasised integrated childhood illness management and nutrition education. FP components included training front-line health workers across settings, group education, risk assessment, individual counselling and same-day contraceptive provision, supplemented in some cases by brochures and educational videos. Referral mechanisms varied; while one study facilitated referrals between FP and immunisation centres, others only allowed referrals from immunisation centres to FP clinics in the same location. Clinic-based services were delivered by vaccinators and counsellors, and community-based activities by front-line health workers.

In a clinic-based integration of FP with child immunisation in Liberia,^48 50^ vaccinators shared brief FP and immunisation messages using a job aid, referring mothers to the colocated FP room for further counselling. The job aid included messages on pregnancy timing, FP benefits for mothers with infants, and immunisation visit reminders. Interested mothers received same-day FP service referral cards and were directed to the FP room; others were given leaflets and encouraged to return.

#### Integration of FP into abortion care

Seven studies focused on integrating FP into abortion care to reduce unsafe abortions and repeated unintended pregnancies, improve reproductive health outcomes, empower women to manage their fertility and lower maternal mortality. None of these studies incorporated a nutritional component into the integrated services. All interventions, except one,^124^ were clinic-based and included staff training, group education, individual counselling, contraceptive provision and referrals. Although all clinic-based studies were conducted in maternal and child health clinics, one study was conducted in the abortion clinic.^44^ The community-based study trained community health workers for outreach and awareness campaigns on postabortion care and FP.^124^ For example, in a clinic-based cluster randomised controlled study in China, two postabortion FP care packages were tested; one package included provider training, information for women and referrals, while the other added extended provider training, individual counselling, free contraceptive materials, male involvement and referrals.^129^

#### Integration of FP through health system strengthening

By strengthening the health system, two studies integrated FP services to enhance provider capacity, ensure commodity supply and improve service quality.^38 47^ One study focused on maternal and child health, upgrading services through provider training and quality improvement. Community activities involved training religious leaders and volunteers for outreach, while system-level efforts included stock management and partnership building.^38^ The other study focused on providing FP within universal healthcare and included a comprehensive range of services including nutrition counselling to group of women and men by a male and female pair of community health workers in villages.^47^

#### Integration of FP into comprehensive sexuality education

Five studies integrating FP into sexuality education aimed to enhance contraception knowledge, provide limited contraceptives and delay marriage and pregnancy.^30 74 87 118 123^ None of these studies included an explicit nutrition component in the service package. Sessions covered contraception, sexual health, gender violence, HIV/AIDS and life skills. Condom provision was universal; one study added pregnancy testing kits. Two school-based studies used trained teachers or peers^87 123^ while two community-based studies used trained peers.^30 118^ One study offered web-based education with private contraceptive access.^74^

### Quality of evidence

Online supplemental file 4 presents the overall methodological quality of the included studies. Of the 109 articles included in the review, 8 were classified as strong in quality, 34 as moderate, and the remaining as weak. Among the reasons for weak quality were: weak research methods and study design such as before-after-studies lacking a control group, failure to consider external factors that could influence the outcomes, failure to report confidence intervals for estimates, inadequate control of confounding factors when estimating the effects, more than 20% dropout rates at follow-up assessments and missing data on components under consideration and poor applicability to other contexts in the country.

### Outcomes reported

#### Quantitative studies

Table 1 summarises intervention effects based on integration types, while online supplemental file 5 details study-specific effects. Among 43 studies examining the integration of FP with packages of maternal and child health services, 26 studies were conducted with control groups, comprising 16 quasi-experimental studies and 10 RCTs. Outcomes reported encompassed FP, maternal and child health, nutrition and SRH. The most common outcome was uptake of modern contraceptives, reported in 22 studies that included control arms like routine maternal and child healthcare or standard care in the health facility. Notably, 72.7% (16 out of 22) of these studies reported increased uptake of modern contraceptives following integration with maternal and child health services. Among these 16 studies, findings from three integrated qualitative studies indicated that offering FP counselling during maternal and child health services, along with involving husbands or other gatekeepers in FP decisions, contributed to a higher uptake of modern contraceptives in intervention groups than in control groups.^63 83 94^ Six studies documented acceptance of implants, with four indicating beneficial effects on birth spacing or effective FP over the desired period. Among five studies integrating FP and sexual health education, only one reported contraception use as an outcome without evidence of effect. No study reported negative impacts on FP outcomes due to integration. Nutrition outcomes were reported sparsely, with the most common being early breastfeeding initiation and exclusive breastfeeding practice.

**Table 1:** Summary of results of integrated family planning (FP) and nutrition or sexual and reproductive health (SRH) services in low‐income and middle‐income countries

Outcome	Integrated interventions*					
	FP and maternal and child health (MCH) intervention (n=43)			FP and other SRH Intervention (n=5)		
	Number of studies assessing outcome	Number of studies with no effect	Number of studies with evidence of effect	Number of studies assessing outcome	Number of studies with no effect	Number of studies with evidence of effect
FP						
Met need for FP	1	–	1	–	–	–
Unmet needs for spacing	1	1	–	–	–	–
Any method of contraception	3	1	2	1	–	1
Uptake of modern contraceptives	22	6	16	–	–	–
Uptake of traditional contraceptives	3	2	1	–	–	–
Time to uptake of modern contraceptive	1	0	1	–	–	–
Use of Lactational amenorrhea method	4	1	3	–	–	–
Use of short acting methods	1	1	–	1		1
Acceptance of condoms	2	0	2	–	–	–
Acceptance/use of oral contraceptive pills	2	0	2	–	–	–
Acceptance of injectables	2	2	0	–	–	–
Acceptance/use of sterilisations	3	2	1	–	–	–
Acceptance/use of intrauterine device	6	3	3	–	–	–
Acceptance/use of implants	3	0	3	–	–	–
Continuation of use of contraceptive pills	1	0	1	–	–	–
Continuation of use of intrauterine device	1	0	1	–	–	–
Birth spacing interval	4	0	4	–	–	–
Desire for pregnancy within 2 years	2	2	0	–	–	–
Knowledge of optimal birth spacing	1	0	1	–	–	–
Willing to use contraceptives	2	2	0	–	–	–
Received FP services	1	1	0	–	–	–
Knowledge of FP methods	6	1	5	–	–	–
Intention to use contraceptives	3	2	1	–	–	–
Subjective norms to use contraceptives	1	–	1	–	–	–
Intention to use LARC method	1	–	1	–	–	–
Joint decision-making of contraceptive use	1	0	1	–	–	–
Spousal agreement on contraception	1	–	1	–	–	–
At-risk of pregnancy	1	0	1	–	–	–
Perceived susceptibility to pregnancy	1	0	1	–	–	–
MCH						
Early breastfeeding initiation	5	2	3	–	–	–
Exclusive breastfeeding practice	5	3	2	–	–	–
Breastfeeding knowledge	1	1	0	–	–	–
Attitude toward breastfeeding	1	0	1	–	–	–
Knowledge of pregnancy and childbirth	1	0	1	–	–	–
Institutional birth rates	2	0	2	–	–	–
Home birth rates	1	1	–	–	–	–
Adolescent pregnancies	1	1	–	–	–	–
Rates of unwanted pregnancies	1	1	–	–	–	–
Rates of repeated abortion	1	1	–	–	–	–
Neonatal mortality	1	1	0	–	–	–
Negative influence of FP on MCH practices	1	1	0	–	–	–
Wrapping infants	1	0	1	–	–	–
Practice of skin-to-skin care	1		1			
Delay bathing for 3 or more days	1		1			
Preterm birth	1	0	1	–	–	–
Infant immunisations	5	3	2	–	–	–
Wasting in children <5 years	1	1	–			
Stunting in children <5 years	1	1	–			
Underweight in children <5 years	1	1	–			
Use of integrated child development services	1	1	–			
SRH						
Knowledge of reproductive health	–	–	–	1	0	1
Sexual health discussions	–	–	–	1	0	1
Attitude towards reproductive health	–	–	–	1	0	1
Perceived safe sex self-efficacy	–	–	–	1	0	1
Sexual activity	–	–	–	1	1	0
Number of partners	–	–	–	1	1	0
Condom use	–	–	–	1	1	0
Early bleeding control	1	–	1			
Post abortion care	1	1	–			
Knowledge of return to fecundity	1	1	–			

* No integrated efforts were identified that directly assessed the impact of combining FP and nutrition interventions.

LARC, Long-acting Reversible Contraception.

Several integrated programmes combining FP with maternal and child health monitored process indicators like the number of healthcare professionals trained in FP, availability of training materials and resources on integrated FP and maternal and child health services, percentage of clients receiving FP counselling during antenatal care or postpartum care visits, percentage of clients receiving their chosen FP method on the same day of counselling, percentage of clients referred from maternal and child health services to specialised FP services for complex needs and acceptance and use of traditional and modern contraceptives. For example, studies that included FP counselling during antenatal or postpartum care visits reported counselling rates ranging from around 30% to as high as 70%. Nutrition indicators were mainly focused on coverage and distribution of services. For example, nutrition counselling coverage within maternal and child health programmes ranged from 20% to 50%, with notable variations in the quality and consistency of counselling provided.

#### Qualitative studies

23 articles explored the perceptions of clients and healthcare providers regarding FP services (table 2). They addressed acceptability, effectiveness, access challenges and service improvement suggestions. Home-based contraceptive delivery alongside maternal and child healthcare reduced barriers for women. Story-based approaches incorporating narratives and storytelling into the counselling process enhanced maternal engagement in counselling. Barriers included women’s decision-making power regarding contraceptive use, husband’s disapproval, stigma and concerns about the side effects of contraceptive methods. Engaging gatekeepers, including community leaders, husbands and in-laws, was identified as an effective strategy to reduce stigma and improve the uptake of contraceptives. Facility-based integration yielded moderate to high client satisfaction. Immunisation-linked FP counselling was well-received, but referrals were inadequate. Lack of privacy and FP counselling in a group setting were identified as potential barriers to integrating FP in the immunisation centres. Integration in school, community and online settings showed promise. Providers appreciated designated counsellor rooms, client-centred care, belief in benefits of FP and birth spacing, workload management, training, consistent supplies and service incentives. However, combining resources was not always perceived as ensuring consistent service delivery.

**Table 2:** Summary results of qualitative evaluations assessing integration of family planning (FP), nutrition and sexual and reproductive health services

Author (year)	Theme	Summary results
Abdel-Tawab *et al*^22^	Acceptability	Women expressed satisfaction with integrated FP counselling during antenatal care in the facilities and during home visits by the community health workers (CHWs) than the women in the control group. Two-thirds of the women in the integrated facilities, half in the integrated community model and less than half in the control arm accepted the postpartum FP methods.
Baqui *et al*^35^	Challenges	Low PPFP use due to religious beliefs, health concerns and husband disapproval.
Cooper *et al*^48^	Challenges	Clients reported long wait times to see the FP provider, unclear directions to the FP room and concerns about privacy as reasons for not accepting an FP referral on the same day. Women who met the FP provider but declined FP methods often cited the need to consult with their partners first, preferring to delay FP initiation until their child was older, or being advised by the FP provider against immediate FP use post partum.
Cooper *et al*^49^	AcceptabilityEffectivenessChallenges	Engaging story-based approach effective. One-third adoption rate of contraceptives. Barriers include lack of self-efficacy, partner opposition. Involving CHWs and engaging partners suggested for improved acceptability.
Cooper *et al*^50^	ChallengesSuggestions	Clients declined FP referrals due to long wait times, unclear pathways and privacy concerns. Those who accepted but did not take contraception immediately cited the need to consult partners, preferring to delay until the baby was older or provider advice to wait post partum. Adding privacy screens in immunisation areas improved referral acceptance and follow-through by reducing stigma and allowing focus on information shared by vaccinators.
Dhital *et al*^56^	Suggestion	Providers requested more counselling materials for better postpartum FP counselling.
Dulli *et al*^59^	AcceptabilitySatisfactionChallenges	Women supported FP integration during infant immunisation, mostly satisfied with services. Nonuse of modern methods due to awaiting menses, fear of side effects and breast feeding.
Erhardt-Ohren *et al*^60^	AcceptabilityChallenges	Positive response to integrated FP group education; confusion over referral process. Side effects and lack of spousal engagement hindered use.
Fotso *et al*^63^	AcceptabilityEffectiveness	Engaging men in MCH care-FP programme boosted contraceptive acceptance. CHWs escorted women to facilities which reduced access barrier.
Hamon *et al*^70^	Challenges	Shortage of contraceptive supplies at the immunisation centres as a major barrier for the utilisation of the services.
Hémono *et al*^74^	AcceptabilityEffectiveness	High acceptance but underutilised services among adolescents.
Hoyt *et al*^77^	EffectivenessChallenges	Women accepted integrated FP and child immunisation services due to convenience in accessing services. Integrated service provided a counselling opportunity for providers. Barriers: fear of side effects, stigma and disapproving husbands.
Jarvis *et al*^83^	AcceptabilityEffectiveness	High uptake of long-acting reversible contraceptive methods when contraceptive methods and postpartum FP counselling provided after delivery in the MCH care. High satisfaction with the services among women receiving integrated services.
Kibel *et al*^85^	EffectivenessChallenges	Home visits by CHWs with pregnancy test kits benefited women facing stigma or travel limitations. Early pregnancy detection aided in antenatal care and FP initiation. Intervention’s success dependent on test kit supply and CHW transportation support. Improved reproductive health choices noted, but not for women seeking abortion.
McPherson *et al*^94^	AcceptabilityEffectivenessChallenges	Use of cards by CHWs during counselling preferred by women and husbands. Card on antenatal care and nutrition cards received good response, request for more information on delivery and FP. Women suggested involvement of husbands and other gatekeepers in FP decision-making.
Nelson *et al*^99^	AcceptabilityChallenges	Low acceptance of FP services during child immunisation visits due to partner opposition, privacy concerns, shyness and method side effects.
Phillips *et al*^104^	ChallengesAcceptability	Barriers to facility care: cultural practices, out-of-pocket payments. Community providers preferred over traditional healers for MCH services, but not community health volunteers.
Puri *et al*^107^	SatisfactionChallenges	Low satisfaction among women receiving FP counselling during antenatal care than women receiving counselling from a dedicated FP provider. Reasons for dissatisfaction with counselling services included a crowded environment, short time with the provider, non-availability of provider, long waiting times, a limited number of days for antenatal care services and lack of comprehensive FP-related information, education and communication materials.
Routh *et al*^109^	AcceptabilityEffectiveness	Participants prefer clinic-based integration of FP services over home visiting in urban areas. Clinic-based model perceived to boost uptake of long-acting contraceptives and overall service utilisation due to convenience.
Scanteianu *et al*^112^	Effectiveness	Providers reported that integrated intervention increased the uptake of FP and MCH services and enhanced provider efficiency through teamwork.
Senderowicz *et al*^114^	Challenges	Postpartum intrauterine device programmes perceived as directive towards postpartum intrauterine device and biased due to the exclusion of other contraceptive methods.
Tebbets and Redwine^118^	AcceptabilityEffectiveness	School and community-based sexual health programme well-received by parents and teens. Teenage pregnancy reduced, peer-led condom distribution valued for contraceptive access and knowledge empowerment.
Wendot *et al*^127^	AcceptabilityEffectiveness	Providers found quality improvement intervention beneficial, enhancing postabortion contraceptive service delivery.

MCH, maternal and child health; PPFP, Postpartum Family Planning.

## Discussion

This scoping review synthesises evidence from 109 articles from 84 studies across 42 LMICs on integrated interventions of FP with nutrition or other SRH services among adolescent girls and women in reproductive ages. Despite significant integration of FP within maternal and child health programmes, the role and impact of nutrition services were often unclear,^131^,^133^ and no standalone integration between FP and nutrition services was found.

Notably, the integration of FP with nutrition programming is largely confined to maternal and child health services and rarely extends to broader life stages or population-based settings such as schools. The linkages between FP and nutrition within the maternal and child health programming were poorly connected, often operating through separate delivery channels with limited coordination of strategies and supplies. The lack of global standards for incorporating nutrition-specific interventions further hinders progress, underscoring the need for clearer guidelines and innovative, cross-sectoral approaches to better reach and support women of reproductive age and their children.^131^

Integrating FP with nutrition and other SRH services typically employed team-based and patient-centric strategies within facility or across service platform referrals, often initiated during the programme design phase due to significant unmet needs for FP.^10^,^1214^ However, wide variation in integration strategies, service platforms, life stages targeted and study designs poses major challenges for analysis and synthesis, further complicated by inconsistent integration terminology and varying implementation methods. These systemic inconsistencies highlight the need for clearer definitions and standardised approaches to guide effective integration and facilitate meaningful comparisons.^11 12 14^

Conversely, integrating FP with abortion services has demonstrated benefits—such as streamlined care, reduced repeat unintended pregnancies and improved convenience through colocated services.^37 43 45 55 127 129^ However, the applicability of these lessons to FP and nutrition integration requires careful consideration. Abortion and contraceptive services share overlapping needs, providers and delivery platforms (often both fall under FP programmes), whereas nutrition services operate within distinct frameworks (eg, maternal–child health, food security). However, insights from FP and abortion integration, such as the value of reducing fragmentation and leveraging existing contact points, may still inform FP and nutrition integration models, provided adaptations are made to account for differences in scope and stakeholder engagement.

Integrated FP services identified here combined education, counselling and the distribution of commodities such as pills and condoms, with additional referrals for long-acting contraceptives offered through health centres. While these services are readily provided by community-based initiatives and clinic-based child immunisation programmes, referral systems often fall short in detail and effectiveness, a challenge echoed in integrated efforts of HIV and FP programmes.^16^ Such deficiencies frequently lead to suboptimal uptake of FP services, underscoring the need for robust health system support and structured service delivery reforms including training, service coordination and enhanced supervision. Furthermore, integrating FP with nutrition within maternal and child health programmes—through education, counselling and behaviour change communication via diverse platforms—demonstrates a holistic approach but requires careful consideration of resource allocation and service quality for creating effective, multifaceted services that address both immediate and long-term needs across integrated domains.^134^

Although methodological variability limited synthesis beyond frequency counts and direction of effects, many studies indicate that integrating FP with nutrition or SRH interventions improves FP outcomes. Most programmes measured at least one FP indicator, with clinic-based studies focusing on service utilisation and community-based studies on contraceptive use. Quantitative evaluations generally assessed the uptake of traditional and modern contraceptives, and there is limited evidence on the effect of these programmes on long-term outcomes such as birth spacing or broader reproductive health needs. Mostly notable gains in nutrition were assessed and reported for children, including improvements in areas such as breastfeeding initiation, reduction in stunting and wasting, and better coverage of vitamin A supplementation. However, no studies disentangled the impact of individual services within the integrated programmes to explore their synergistic and interrelated benefits, such as how enhanced nutrition might improve pregnancy outcomes—a key goal of FP. Several process evaluations monitored integration metrics like healthcare training and contraceptive distribution and acceptance, but rarely reported the quality of integrated services compared with siloed approaches, which can provide insights into the interactions between services and potential attribution pathways. Finally, these studies rarely assessed cost-effectiveness, revealing a gap in data on efficiency and associated costs and underscoring the necessity for economic evaluations before broader adoption of integration strategies.^11 134^

Qualitative evaluation highlighted the acceptability and perceived relevance of FP services among clients, along with the feasibility of delivering these services within broader programmes. Few studies, however, addressed provider-related factors, including staff motivation, beliefs, attitudes, incentives, training needs and ongoing support. Despite limited data, most providers, clients and community members viewed integration efforts positively. Given the limited studies reporting implementation indicators specific to integration of FP with nutrition and other services, it is crucial to assess a broad range of indicators, including cost-effectiveness, adoption, sustainability and transferability. Future studies should also explore stakeholder perspectives on integrated services, including feasibility and acceptability conditions (eg, fixed locations vs outreach sessions), cost mitigation and strategies for enhancing the integration of clinic-based and community-based services.

Despite these potentials, notable challenges and evidence gaps persist in the evidence. The quality of evidence is a major concern, with only 8 out of 109 studies rated as strong and just one-third of studies demonstrating moderate to strong design. While strategies like home-based contraceptive delivery coupled with facility referrals have shown promise in reducing barriers, issues like stigma, husband’s disapproval and privacy concerns persist. The effectiveness of these integration strategies is further influenced by local cultural dynamics and health system capacities, which can vary widely between settings. This review is the first systematic synthesis of both published and grey literature on the integration of FP with nutrition and other services. However, it is limited by its focus on English-language literature and the broad nature of scoping reviews, which may introduce bias by excluding relevant studies published in other languages, particularly from LMICs. We mitigated potential bias by searching LMIC-focused literature on relevant websites (eg, Google Scholar) and grey literature from international organisations working in non-English-speaking regions (eg, WHO and UNICEF reports from Francophone Africa). While this decision was pragmatic due to resource constraints, future reviews should prioritise multilingual searches to capture a more comprehensive evidence base.

Our review identified key facilitators and barriers to integrating FP with nutrition or SRH services, emphasising the importance of strong community networks and existing programme infrastructures supported by national or subnational policies and aligning with existing evidence.^8 11 12 15 16 135^ These elements are crucial for expanding services, reducing costs and building trust. Using community structures, such as village health or market days and targeting the 1000 day period through a continuum of care model are effective strategies to meet the needs of women, children and families.^8 11 12 15^ Tailored messaging, home visits and consistent contact at community and facility levels enhance family engagement and ensure uniform messaging, while engaging gatekeepers like religious leaders, husbands and in-laws helps overcome barriers to uptake.^11 15 16 135^ However, challenges like staffing shortages, volunteer incentives and the consistent supply of FP commodities require urgent attention.^11 14^ Our findings suggest that policy-makers and programme designers must adapt integration models to local contexts, considering cultural norms, health infrastructures and policy environments. Training and supporting healthcare workers are critical to success, underscoring the need for investment in human resources alongside materials and infrastructure. Future research should focus on longitudinal studies to understand the long-term effects of integrating FP with nutrition and SRH services, improve research quality through robust study designs, and explore the economic impacts of integrated service delivery, particularly in resource-limited settings. These recommendations emerge not from proven effectiveness, but from recurrent implementation barriers observed across studies. The absence of cost-effectiveness data does not negate integration’s value, but signals critical evidence gaps for policy-makers.

In conclusion, we found a small evidence base detailing the integration of FP with nutrition services, with relatively more research documenting the integration of FP and SRH services. Rather than re-establishing the feasibility of FP and SRH integration, our review highlights its uneven application across SRH domains, transferable strategies for FP and nutrition and systemic barriers threatening all integration types. The available data reinforce the idea that integrating FP with nutrition services holds considerable promise for enhancing health outcomes among women of reproductive age and their children. However, the approach requires careful adaptation to local contexts, robust methodological frameworks and sustained resource allocation to overcome existing barriers and maximise its potential benefits. Further, more streamlined and consistent design and documentation of such efforts will be crucial to more clearly establish the feasibility and value of integrating FP and nutrition services.

## Supplementary material

10.1136/bmjgh-2024-017482 online supplemental file 1

## Data Availability

All data relevant to the study are included in the article or uploaded as supplementary information.
