# Supplementary material for: Integrating family planning with nutrition and other sexual and reproductive health services in low-income and middle-income countries: findings from a scoping review
Source: BMJ Glob Health. 2025 May 30;10(Suppl 1):e017482. doi: 10.1136/bmjgh-2024-017482 (PMC12128479; doi:10.1136/bmjgh-2024-017482)
Supplement: online supplemental file 1 [file bmjgh-10-Suppl_1-s001.pdf]

Supplementary files
Online Supplementary File 1: PubMed search strategy

No.	Concept	PubMed search terms	Number of records (As of 16 February 2023)
#1	**Randomized controlled trial**<br>**Controlled before-after studies**<br>**Quasi experimental studies**<br>**Qualitative studies**<br>**Case study**	("randomized controlled trial"[Publication Type] OR "randomized controlled trials as topic"[MeSH Terms] OR "randomized controlled trial"[All Fields] OR "randomised controlled trial"[All Fields]) OR ("double blind method"[MeSH Terms] OR ("double blind"[All Fields] AND "method"[All Fields]) OR "double blind method"[All Fields] OR ("double"[All Fields] AND "blind"[All Fields]) OR "double blind"[All Fields]) OR ("single blind method"[MeSH Terms] OR ("single blind"[All Fields] AND "method"[All Fields]) OR "single blind method"[All Fields] OR ("single"[All Fields] AND "blind"[All Fields] AND "method"[All Fields]) OR "single blind method"[All Fields]) OR ("clinical trial"[Publication Type] OR "clinical trials as topic"[MeSH Terms] OR "clinical trial"[All Fields]) OR ("comparative study"[Publication Type] OR "comparative study"[All Fields]) OR ("before-after"[All Fields] AND ("studies"[All Fields] OR "study"[All Fields] OR "study s"[All Fields] OR "studying"[All Fields] OR "studys"[All Fields])) OR ("quasi"[All Fields] AND ("studies"[All Fields] OR "study"[All Fields] OR "study s"[All Fields] OR "studying"[All Fields] OR "studys"[All Fields])) OR ("evaluation study"[Publication Type] OR "evaluation studies as topic"[MeSH Terms] OR "qualitative evaluation"[All Fields]) OR ("case reports"[Publication Type] OR "case study"[All Fields])	6,594,851
#2	**Adolescent girls and women**	("Women"[Mesh] OR women[tiab] OR woman[tiab] OR "Adolescent"[Mesh] OR Adolescent*[tiab] OR teen[tiab] OR teens[tiab] OR teenager*[tiab] OR girl*[tiab] OR "Mothers"[Mesh] OR mother*[tiab])	3,715,590
#3	**Low- and middle-income countries**	("Developing Countries"[MeSH] OR "developing countr*"[tiab] OR "developing nation*"[tiab] OR "less developed countr*"[tiab] OR "less developed nation*"[tiab] OR "third world nation*"[tiab] OR "third world countr*"[tiab] OR "under developed nation*"[tiab] OR "underdeveloped nation*"[tiab] OR "under developed countr*"[tiab] OR "underdeveloped countr*"[tiab] OR "middle income countr*"[tiab] OR "middle-income countr*"[tiab] OR "middle income nation*"[tiab] OR "middle-income nation*"[tiab] OR "low income countr*"[tiab] OR "low-income countr*"[tiab] OR "low income nation*"[tiab] OR "low-income nation*"[tiab] OR "poor countr*"[tiab] OR "poor nation*"[tiab] OR LMIC[tiab] OR LMICs[tiab] OR "Africa"[MeSH] OR "Asia"[MeSH] OR "South America"[MeSH] OR "Latin America"[MeSH] OR "Central America"[MeSH] OR africa[tiab] OR asia[tiab] OR "south america*"[tiab] OR "latin america*"[tiab] OR "central america*"[tiab] OR Afghanistan*[tiab] OR Albania*[tiab] OR Algeria*[tiab] OR Samoa*[tiab] OR Angola*[tiab] OR Armenia*[tiab] OR Azerbaijan*[tiab] OR Bangladesh*[tiab] OR Bengali[tiab] OR Belarus*[tiab] OR Belize[tiab] OR Benin[tiab] OR Bhutan*[tiab] OR Bolivia*[tiab] OR Bosnia*[tiab] OR Herzegovina*[tiab] OR Botswana*[tiab] OR Brazil*[tiab] OR Bulgaria*[tiab] OR "Burkina Faso"[tiab] OR Burkinabe[tiab] OR Burundi*[tiab] OR "Cabo Verd*"[tiab] OR "Cape Verd*"[tiab] OR Cambodia*[tiab] OR Cameroon*[tiab] OR "Central African*"[tiab] OR Chad*[tiab] OR China[tiab] OR Chinese[tiab] OR Colombia*[tiab] OR Comoros[tiab] OR Congo[tiab] OR "Costa Rica*"[tiab] OR "Cote d'Ivoire"[tiab] OR "Ivory Coast"[tiab] OR Cuba[tiab] OR Cuban[tiab] OR Djibouti[tiab] OR Dominica*[tiab] OR Ecuador[tiab] OR Egypt*[tiab] OR "El Salvador*"[tiab] OR Eritrea*[tiab] OR Ethiopia*[tiab] OR Fiji*[tiab] OR Gabon*[tiab] OR Gambia*[tiab] OR Georgia*[tiab] OR Ghana*[tiab] OR Grenada*[tiab] OR Guatemala*[tiab] OR	2,635,737

		Guinea*[tiab] OR Guyan*[tiab] OR Haiti*[tiab] OR Hondura*[tiab] OR India[tiab] OR Indian*[tiab] OR Indonesia*[tiab] OR Iran*[tiab] OR Iraq*[tiab] OR Jamaica*[tiab] OR Jordan*[tiab] OR Kazakh*[tiab] OR Kenya*[tiab] OR Kiribati[tiab] OR “People's Republic of Korea”[tiab] OR “North Korea”[tiab] OR Kosovo[tiab] OR Kosovar*[tiab] OR Kyrgyz*[tiab] OR Lao[tiab] OR Laos[tiab] OR Laotian*[tiab] OR Lebanon[tiab] OR Lebanes*[tiab] OR Lesotho[tiab] OR Liberia*[tiab] OR Libya*[tiab] OR Macedonia*[tiab] OR Madagascar*[tiab] OR Malawi*[tiab] OR Malaysia*[tiab] OR Maldives[tiab] OR Mali[tiab] OR “Marshall Island*”[tiab] OR “Mexico”[MeSH] OR Mexico[tiab] OR Mexican*[tiab] OR Micronesia*[tiab] OR Moldova*[tiab] OR Mongolia*[tiab] OR Montenegr*[tiab] OR Morocc*[tiab] OR Mozambique[tiab] OR Myanmar[tiab] OR Burmese*[tiab] OR Burma[tiab] OR Namibia*[tiab] OR Nepal*[tiab] OR Nicaragua*[tiab] OR Niger*[tiab] OR Pakistan*[tiab] OR Paraguay*[tiab] OR Peru*[tiab] OR Philippin*[tiab] OR Rwanda*[tiab] OR “Sao Tome”[tiab] OR Principe[tiab] OR Senegal*[tiab] OR Serbia*[tiab] OR “Sierra Leone*”[tiab] OR “Solomon Island*”[tiab] OR Somalia*[tiab] OR “South Africa*”[tiab] OR “Sri Lanka”[tiab] OR “St Lucia”[tiab] OR “Saint Lucia”[tiab] OR “St Vincent”[tiab] OR “Saint Vincent”[tiab] OR Grenad*[tiab] OR Sudan*[tiab] OR Suriname*[tiab] OR Swaziland*[tiab] OR Eswatini*[tiab] OR Syria*[tiab] OR Tajik*[tiab] OR Tanzania*[tiab] OR Zanzibar[tiab] OR Thai*[tiab] OR Timor*[tiab] OR Togo*[tiab] OR Tonga*[tiab] OR Tunisia*[tiab] OR Turkey[tiab] OR Turkish[tiab] OR Turkmen*[tiab] OR Tuvalu*[tiab] OR Uganda*[tiab] OR Ukrain*[tiab] OR Uzbeki*[tiab] OR Vanuatu*[tiab] OR Venezuela*[tiab] OR Vietnam*[tiab] OR “Viet nam*”[tiab] OR “West Bank”[tiab] OR Gaza*[tiab] OR Palestin*[tiab] OR Yemen*[tiab] OR Zambia*[tiab] OR Zimbabw*[tiab] OR “Western Sahara”[tiab] OR Argentin*[tiab] OR Russia*[tiab] OR Maurit*[tiab] OR Palau[tiab])	
#4	**Integrated interventions /services /clinics /programs**	("integrability"[All Fields] OR "integrable"[All Fields] OR "integral"[All Fields] OR "integrally"[All Fields] OR "integrals"[All Fields] OR "integrant"[All Fields] OR "integrants"[All Fields] OR "integrate"[All Fields] OR "integrated"[All Fields] OR "integrates"[All Fields] OR "integrating"[All Fields] OR "integration"[All Fields] OR "integrational"[All Fields] OR "integrations"[All Fields] OR "integrative"[All Fields] OR "integratively"[All Fields] OR "integrator"[All Fields] OR "integrators"[All Fields])	981,993
#5	**Nutrition**	"nutrition programme"[All Fields] OR "health planning"[MeSH Terms] OR ("health"[All Fields] AND "planning"[All Fields]) OR "health planning"[All Fields] OR ("nutrition"[All Fields] AND "program"[All Fields]) OR "nutrition program"[All Fields] OR ("diet therapy"[MeSH Terms] OR ("diet"[All Fields] AND "therapy"[All Fields]) OR "diet therapy"[All Fields] OR ("nutrition"[All Fields] AND "intervention"[All Fields]) OR "nutrition intervention"[All Fields]) OR ("dietary supplements"[MeSH Terms] OR ("dietary"[All Fields] AND "supplements"[All Fields]) OR "dietary supplements"[All Fields] OR ("nutrient"[All Fields] AND "supplementation"[All Fields]) OR "nutrient supplementation"[All Fields]) OR (("food"[MeSH Terms] OR "food"[All Fields]) AND ("fortification"[All Fields] OR "fortifications"[All Fields])) OR (("weight s"[All Fields] OR "weighted"[All Fields] OR "weighting"[All Fields] OR "weightings"[All Fields] OR "weights and measures"[MeSH Terms] OR ("weights"[All Fields] AND "measures"[All Fields]) OR "weights and measures"[All Fields] OR "weight"[All Fields] OR "body weight"[MeSH Terms] OR ("body"[All Fields] AND "weight"[All Fields]) OR "body weight"[All Fields] OR "weights"[All Fields]) AND ("intervention s"[All Fields] OR "interventions"[All Fields] OR "interventive"[All Fields] OR "methods"[MeSH Terms] OR "methods"[All Fields] OR "intervention"[All Fields] OR "interventional"[All Fields]))	1,527,469
#6	**Family Planning**	contracepti*[tiab] OR family planning[tiab] OR family planning[mh] OR birth control[tiab] OR birth control[mh] OR contraception injectable*[tiab] OR IUD[tiab] OR IUCD[tiab] OR IUS OR intrauterine device*[tiab] OR intra-uterine device*[tiab] OR intrauterine system*[tiab] OR intra-uterine system*[tiab] OR oral contraceptive pill*[tiab] OR hormonal contraceptive pill*[tiab] OR birth control pill[tiab] OR emergency contraceptive pill*[tiab] OR cervical cap*[tiab] OR	740,909

		vaginal diaphragm*[tiab] OR vaginal ring*[tiab] OR implant*[tiab] OR subdermal implant*[tiab] OR implanon[tiab] OR jadelle[tiab] OR norplant*[tiab] OR sino-implant*[tiab] OR sinoimplant*[tiab] OR injectable*[tiab] OR contraceptive patch[tiab] OR sterilization[tiab] OR sterilisation[tiab] OR vasectomy[tiab] OR contraception behaviour[tiab] OR long-acting reversible contraception[tiab] OR family planning[mh] OR birth control[mh] OR contraception[mh] OR Family Planning Services[mh] OR "sterilization, reproductive"[MeSH Terms] OR lactational amenorrhea method [tiab] OR lactational amenorrhoea method[tiab] OR sympto-thermal method[tiab] OR twoday method[tiab] OR standard days method[tiab] OR basal body temperature method[tiab] OR rhythm method[tiab] OR calendar method[tiab] OR condom*[tiab] OR withdrawal[tiab] OR coitus interruptus[tiab]	
#7	**Sexual and Reproductive health**	((("sexual health"[MeSH Terms] OR ("sexual"[All Fields] AND "health"[All Fields]) OR "sexual health"[All Fields] OR ("reproductive health"[MeSH Terms] OR ("reproductive"[All Fields] AND "health"[All Fields]) OR "reproductive health"[All Fields])) AND ("program"[All Fields] OR "program s"[All Fields] OR "programe"[All Fields] OR "programed"[All Fields] OR "programes"[All Fields] OR "programing"[All Fields] OR "programmability"[All Fields] OR "programmable"[All Fields] OR "programmably"[All Fields] OR "programme"[All Fields] OR "programme s"[All Fields] OR "programmed"[All Fields] OR "programmer"[All Fields] OR "programmer s"[All Fields] OR "programmers"[All Fields] OR "programmes"[All Fields] OR "programming"[All Fields] OR "programmings"[All Fields] OR "programs"[All Fields])) OR ("sexual health"[MeSH Terms] OR ("sexual"[All Fields] AND "health"[All Fields]) OR "sexual health"[All Fields] OR ("reproductive health"[MeSH Terms] OR ("reproductive"[All Fields] AND "health"[All Fields]) OR "reproductive health"[All Fields]))) AND ("intervention s"[All Fields] OR "interventions"[All Fields] OR "interventive"[All Fields] OR "methods"[MeSH Terms] OR "methods"[All Fields] OR "intervention"[All Fields] OR "interventional"[All Fields])	1118,682
A	**Study type, population, LMIC, nutrition, family planning**		
	(#1) AND (#2) AND (#3) AND (#5) AND (#6)		2,947
B	**Study type, population, LMIC, family planning, sexual and reproductive health**		
	(#1) AND (#2) AND (#3) AND (#6) AND (#7)		1,764
C	**Study type, population, LMIC, integration**		
	(#1) AND (#2) AND (#3) AND (#4)		3,707
D	**Search A, limited by language and publication date**		
	(#1) AND (#2) AND (#3) AND (#5) AND (#6), English, 2000 onwards		1,162
E	**Search B limited by language and publication date**		
	(#1) AND (#2) AND (#3) AND (#6) AND (#7) English, 2000 onwards		1,275
F	**Search C limited by language and publication date**		
	(#1) AND (#2) AND (#3) AND (#4), English, 2000 onwards		2,985
	**Combination of D + E + F**		**5,422**

Online Supplementary File 2: Reflexivity Statement

1. How does this study address local research and policy priorities?

This study synthesizes evidence from low- and middle-income countries (LMICs), particularly sub-Saharan Africa and South Asia, focusing on gaps and opportunities for integrating family planning, nutrition, and other sexual and reproductive health services. The findings aim to inform local policies and interventions, addressing a critical area of concern identified in previous research.

2. How were local researchers involved in study design?

The local researchers involved bring extensive scoping review experience. SS, UP, and NA, originally from LMICs but now based in high-income countries, have a strong background in local research within international collaborations. MO and ISY have significant public health research expertise in Burkina Faso and Tanzania, respectively. CAY specializes in maternal and child nutrition and reviews. WF and IS have extensive experience in leading international research collaborations involving LMICs. All authors contributed to the development and refinement of this review. Hailing from diverse cultural backgrounds, we are committed to addressing inequities and promoting equitable research practices.

3. How has funding been used to support the local research team?

Funding has supported the local research team by covering training costs for conducting scoping reviews. It has also facilitated a workshop in Dar es Salaam, bringing together local researchers, policymakers, and program leaders (including international organizations in Tanzania) to present preliminary findings from this review and other related studies. This workshop helped expand scientific and programmatic networks and strengthened partnerships between local research teams and key local stakeholders.

4. How are research staff who conducted data collection acknowledged?

SS, CAY, and NA designed the review protocol with input from all authors. They also led the drafting of the search strategy, conducted searches in selected databases, and handled screening and data extraction. All authors contributed to data analysis and interpretation, with each team member assigned specific roles during the writing process.

5. Do all members of the research partnership have access to study data?

Yes, all research team members have access to study data.

6. How was data used to develop analytical skills within the partnership?

All the authors listed on this manuscript in all the stages of research, with intentions to utilize multidisciplinary skills and develop accessible language for a broad research audience.

7. How have research partners collaborated in interpreting study data?

Three workshops were held during the analysis stage. The first workshop focused on familiarizing researchers with the data and data extraction sheets. Researchers then formed working groups for analysis and interpretation, followed by a collective discussion of findings. These were presented at a subsequent workshop, where recommendations and reflexivity statements were finalized collaboratively.

8. How were research partners supported to develop writing skills?

The research team drafting this statement is primarily made up of senior academics. Early career researchers (SS, CAY, NA, UP, MO, and ISY) received support from senior academics (IS and WF) to develop and refine their writing skills.

9. How will research products be shared to address local needs?

This manuscript will be published as part of a Special Issue on integrating family planning and nutrition services, and will be open access. We plan to widely disseminate the issue, reaching global health leaders, international collaboration experts, and stakeholders in both high- and low- and middle-income countries.

10. How is the leadership, contribution and ownership of this work by LMIC researchers recognised within the authorship?

SS, originally from an LMIC, contributed as the first author in developing this manuscript, collaborating closely with the senior authors. CAY, from a high-income country, and NA, also from an LMIC, made significant contributions as co-authors. The authorship team includes researchers from and based in LMICs (UP, MO, and ISY). However, the team is primarily composed of individuals based in high-income countries, as the initiative was led by a principal investigator based in a high-income country.

11. **How have early career researchers across the partnership been included within the authorship team?**
We have included early career researchers (SS, CAY, NA, UP, MO, and ISY) within the authorship team. They contributed to multiple stages of this research.

12. How has gender balance been addressed within the authorship?

Five authors are male (SS, MO, ISY, IS and WF) and three authors female (CAY, NA, and UP); all the female authors were actively engaged in conceiving the study, data collection, data analysis and interpretation.

13. How has the project contributed to training of LMIC researchers?

The authorship team includes both early-career and senior researchers. Research funding supported the employment of a junior researcher in Tanzania and an early-career researcher in Burkina Faso.

14. How has the project contributed to improvements in local infrastructure?

This project has not directly contributed to improvements in local infrastructure.

15. What safeguarding procedures were used to protect local study participants and researchers?

Being a scoping review, there were no study participants in this study.

Online Supplementary File 3: Characteristics of included studies

Author, Year and Country	Study Type	Study methods	Population	Intervention	Comparison	Findings
Abdel-Tawab 2008, Egypt[20]	Evaluation study	(1) Exit and home interviews with low parity pregnant women at 4 months and 10-11 months postpartum; (2) structured interviews with healthcare providers; (3) in-depth interviews with healthcare supervisors; (4) focus group discussions (FGDs) with community members; (5) FGDs with husbands of low parity women; and (6) service statistics	Pregnant women, their husband	Family planning (FP) services: (1) A protocol for birth spacing messages to pregnant and postpartum women through antenatal care and postpartum care, (2) IEC materials for women, men, and health care providers, and (3) awareness-raising activities for men through community seminars, one-to-one meetings, informal discussions<br><br>Model I: 1 and 2 components<br>Model II: all three components<br><br>Maternal and child health (MCH) services: Comprehensive MCH services including nutrition counseling and quarterly home visits to educate women about mother and infant nutrition and health<br><br>Integration point: MCH-FP services included for provision by doctors, nurses, and community health workers in the health facility and community settings	Standard care only	Providing birth spacing messages to low parity women during antenatal and postpartum care and to husbands through community awareness activities was feasible and acceptable<br><br>Both models effective in changing women's knowledge and attitudes towards birth spacing and in enhancing use of contraception at 10-11 months postpartum, by 48% among Model I mothers and 43% among Model II mothers, compared with 31% among control group mothers
Abdel-Tawab 2011, Egypt[21]	Operation research	Service statistics	Pregnant and postpartum women	FP services: (1) Revising and updating birth spacing messages protocol for pregnant and postpartum women; (2) training of trainers for FP and MCH managers and supervisors; (3) on the job training of clinic staff (doctors, nurses and community health workers; (4) providing information, education and communication (IEC) materials for clients and job aids for providers; (5) monitoring and supervision; (6) seminars for husbands; (7) steering	None	Successful scaling up of the intervention in a total of eight districts of two governorates and created conditions for scaling up in remaining 16 districts of these two governorates<br><br>Secured support and investment from Ministry of Health and Population for future scale up

				committee meetings; and (8) a national orientation and dissemination workshop<br><br>MCH services: Comprehensive maternal and child health services including nutrition education to mothers<br><br>Integration point: FP services were included for provision by doctors, nurses, and community health workers in the health facility and community settings		
Abdulahi 2021, Ethiopia[22]	Clustered randomized controlled trial	Interviews at baseline, 1- and 6-months postpartum	Pregnant women	FP services: Breastfeeding education and support intervention with two strategies: (1) Training of peer supporters, and (2) breastfeeding education and peer support through pre-specified schedule of home visits by peer educators. Along with breastfeeding support intervention, peer educators also discussed the lactation amenorrhea method, and other family planning options in their regular visits<br><br>Nutrition services: nutrition messages to mothers during monthly growth monitoring sessions or during antenatal care (ANC) and postpartum care (PNC) visits<br><br>Integration point: Integrated MCH-FP services at community level	Routine care	Compared to the control, the intervention significantly increased early initiation by 25.9% and exclusive breastfeeding by 14.6% practices
Achyut 2016a, India [23]	Evaluation study	Midline survey	Women who had delivered a live birth	FP services: (1) Provision of postpartum and postabortion FP, (2) training providers to improve technical competence and client–provider interactions, (3) expanding the role of the private sector in FP service provision, (4) using community health workers for outreach efforts and limited FP service provision, and (5) using mid- and mass-media to promote demand for FP services	None	FP information provision as part of antenatal care in the third trimester, delivery, and the postpartum period had a positive association with postpartum modern contraceptive use<br><br>Health providers often miss FP services provision opportunities. Despite a high proportion of women encountering health providers when utilizing MH services, only a small

				MCH services: Regular antenatal, delivery and post-natal services in the existing MCH facilities; nutrition education during regular visits<br><br>Integration point: FP services integrated into MCH through client outreach, social marketing, and media campaign.		proportion received FP information during these interactions
Achyut 2016b, India[24]	Evaluation study	Baseline and endline surveys with women and health facility staff	Women who had delivered a live birth	FP services: (1) Provision of postpartum and postabortion FP, (2) training providers to improve technical competence and client–provider interactions, (3) expanding the role of the private sector in FP service provision, (4) using community health workers for outreach efforts and limited FP service provision, and (5) using mid- and mass-media to promote demand for FP services<br><br>MCH services: Regular antenatal, delivery and post-natal services in the existing MCH facilities; nutrition education during regular visits<br><br>Integration point: FP services integrated into MCH through client outreach, social marketing, and media campaign	None	Women exposed to brochures, billboards/posters/wall hangings, FP on the television, being exposed to community health worker and living close to an improved public and private supply environment where project activities undertook significantly more likely to be using a modern method at endline
Adanikin 2013, Nigeria[25]	Post-intervention assessment	Six-months postpartum interviews with women	Pregnant women	FP services: 'Antenatal group' received one-to-one antenatal contraceptive counselling on several occasions while the 'Postnatal group' received a single one-to-one contraceptive counselling session at the six-week postnatal check; women in both groups received tailored guidance on contraceptive needs along with regular maternal and child health services<br><br>MCH services: Regular antenatal and postnatal services including nutrition education	None	More women who had multiple antenatal contraceptive counselling sessions used modern contraceptive methods than those who had a single postnatal counselling session (57% vs. 35%)

				Integration point: Inclusion of FP services during maternal care visits by the healthcare providers at the health facility		
Ahmed 2013, Bangladesh[26]	Quasi-experimental study	Pre- and post-intervention household surveys	Pregnant and postpartum women	FP services: Use of lactational amenorrhea method (LAM) and modern contraceptive methods to achieve a 24-month birth to pregnancy interval using provision of supplies and counselling and active referrals<br><br>MCH services: Within the maternal and newborn health program: Community health workers (CHWs) trained to provide communication and behavior change, clinical assessment of neonates, and clinical care, included nutrition education to improve maternal and child nutrition status<br><br>Integration point: FP services introduced to the existing MNH program and delivered by trained CHW; FP provision services linked with the facility	Standard maternal and newborn care promotion	Women and their family members generally did not perceive birth spacing as a priority, and most recently delivered women were not using contraception<br><br>CHWs visited over 90% of women in both intervention and comparison groups during pregnancy and the first 3 months postpartum
Ahmed 2015, Bangladesh[27]	Quasi-experimental study	Operations research, survey rounds conducted at baseline (antenatal) and at 3, 6, 12, 18, and 24 months	Pregnant and postpartum women	FP services: (1) Behavior change communication messages related to postpartum FP added to the existing MCH intervention package for interpersonal counseling and group meetings (2) oral contraceptives and condoms provided by community health workers, and (3) monthly meetings organized by community mobilizers for men and women to discuss the importance of pregnancy spacing and postpartum FP, including LAM<br><br>MCH services: Importance of exclusive breast feeding and nutrition education to mothers<br><br>Integration point: Community health workers visited pregnant and postpartum	Maternal and newborn-care services only	Contraceptive prevalence rate (CPR) in the intervention arm was 15% points higher than in the control arm at 12 months, and the difference in CPRs remained statistically significant throughout the 24 months of observation<br><br>The short birth interval of less than 24 months was significantly lower in the intervention arm

				women as a part of existing MCH program; FP provision services linked with the facility		
Ajuwon 2007, Nigeria[28]	Quasi-experimental study	Pre- and post-intervention surveys	Secondary school students	Three models of comprehensive sexuality education with contraception (condom provision)<br>Model I: trained teachers<br>Model II: trained peers<br>Model III: trained teachers and peers<br>Topics covered: adolescent sexuality, communication, human reproduction, pregnancy prevention, sexually transmitted diseases, HIV/AIDS, condom promotion and distribution, drug abuse, etc.<br><br>Integration point: Adolescent sexuality education models covered contraceptive promotion and distribution	No intervention	Reported condom use significantly higher among peer-led group (from 16.7 % to 62.8%) and teacher- and peer-led group (from 22.8% to 53%) compared to only teacher-led group (28.6% to 47.4%) and control (from 25% to 45.8%)<br><br>Students that received education from both teachers and peer educators showed most improvement in knowledge and perceived self-efficacy
Ayiasi 2015, Uganda[29]	Clustered randomized controlled trial	Post-intervention assessment (12-14 months postpartum interviews)	Pregnant women	FP service: Counseling during antenatal period on postpartum contraceptive use<br><br>MCH services: nutrition education as part of antenatal care visits<br><br>Integration point: Village health teams made home visits to promote postpartum contraceptive use as a part of regular antenatal care visits	Standard care including group health education on general maternal and newborn issues offered during antenatal care visits	About 80% initiated breastfeeding within six hours of delivery; 78.4% (control) and 80.4% (intervention). About half of the mothers in each arm had considered delaying the next pregnancy; 47.1% (control) and 49% (intervention). Of these 71.4% in the control and 87% in the intervention had considered to use a modern contraceptive method, only 28.2% of the control and 31.6% in the intervention were current modern contraceptive users
Babalola 2001, Cameroon[30]	Evaluation study	Pre- and post-intervention surveys	Men and women aged 15–49 years	FP services: Community mobilizing activities including formal presentations, groups discussions, music, theater, and dance, focusing on FP, child health and immunization, treatment for common childhood diseases, and HIV and other sexually transmitted disease prevention.	None	Mixed influenced of the program on contraceptive use; impact varied by project sites, which were separated by urban versus rural living

				MCH services: Education on child nutrition, child diseases and Oral Rehydration Solution to community mobilizers<br><br>Integration point: Implementation of FP and child health related mobilization activities through community mobilizers		
Balasubramaniam 2018, India[31]	Quasi-experimental study	Pre- and post-intervention exist interviews with women and service statistics	Postpartum women	FP services: Screening tool to identify women with unmet FP needs; Received FP methods on the screening day or referrals to link to additional resources.<br><br>MCH services: Unmet needs of antenatal care, postnatal care or immunization services were identified, and provided on the spot, or referrals made. Group health and nutrition education through village health and nutrition days<br><br>Integration point: CHWs conducted monthly village health and nutrition days at health centers	CHWs received the same training as intervention group but did not use the screening tool	More women in the intervention group received FP services but no difference was found in the uptake of services<br><br>FP acceptance and FP method distribution increased
Bang 2018, Ethiopia[32]	Evaluation study	Pre- and post-intervention assessment	Women of reproductive age	FP services: (1) Village level and small group education sessions on maternal health education including family planning, (2) encouraging paternal participation in family planning and, (3) one education session was also given to male community leaders.<br><br>MCH services: Village level and small group education sessions included safe messages on health and nutrition during antenatal care, delivery, and postpartum care visits. Mass media was also used to improve awareness of maternal health. T-shirts and calendars carrying maternal health information were also offered to the women and their family in the intervention area	Usual maternal health care plus maternal health information via radio broadcasting and on-the-job training for health-care professionals	Significant increases in knowledge and behaviors regarding maternal health and family planning compared to the comparison group ($p < .001$)<br><br>Significant increase from 10.8% to 93.5% in the institutional birth rate in the intervention group<br><br>Contraceptive use increased from 31.3% to 61.8% in the intervention group and from 33.0% to 35.3% in the comparison group

				Integration point: On-the-job training sessions for various groups of health-care professionals, including nurses, basic emergency obstetric care nurses, midwives, and health extension workers		
Baqui 2011, Bangladesh[33]	Quasi-experimental study	3-, 6-, 12-, and 18-months postpartum assessments	Pregnant and postpartum women	FP Services: Along with regular surveillance visits, CHWs carried out 2 additional visits during postpartum to provide behavior change communication messages and contraceptive provision (pills and condoms) and organize community level meetings to discuss birth spacing during postpartum period including LAM<br><br>MCH services: CHWs were trained in maternal and newborn health (including nutrition messages) and provided key essential newborn care drugs and supplies<br><br>Integration point: Community health workers visited pregnant and postpartum women as a part of existing Health Fertility Study; FP provision services linked with the facility	Maternal and newborn-care services only	Overall, any contraceptive method use at 36 months postpartum was higher among intervention area participants (45%), compared to those in the comparison arm (39%)<br><br>In intervention arm 40% of women became pregnant by 24 months, compared to 48% in the control area. By 36 months postpartum, 47% of women in the intervention area became pregnant compared to 56% in the control area
Baqui 2018, Bangladesh[34]	Quasi-experimental study	24 months postpartum assessment	Pregnant and postpartum women	FP services: Along with regular surveillance visits community health workers carried out to additional visits during postpartum to provide behavior change communication messages and contraceptive provision (pills and condoms) and organize community level meetings to discuss birth spacing during postpartum period including LAM<br><br>MCH services: CHWs were trained in maternal and newborn health (including nutrition messages) and provided key essential newborn care drugs and supplies<br><br>Integration point: Community health workers visited pregnant and postpartum	Maternal and newborn-care services only	Compared to the control arm, women in the intervention arm had a 19% lower risk of short birth interval (adjusted relative risk (RR) = 0.81, 95% confidence interval (CI) = 0.69-0.95) and 21% lower risk of preterm birth (adjusted RR = 0.79; 95% CI = 0.63-0.99)

				women as a part of existing Health Fertility Study; FP provision services linked with the facility		
Baynes 2022, Tanzania[35]	Evaluation study	Pre- and post-training assessments (exit interviews with clients in public facilities and service data)	Women who availed abortion care	FP services: Ministry of Health, Community Development, Gender, Elderly, and Children implemented technical trainings in post-abortion care, focusing on reinforcing the skills of mid-level providers (i.e., midwives and nurses) in FP counseling and insertion and removal of intra uterine devices (IUDs) and implants<br><br>Post-abortion care: decentralization of post-abortion care from tertiary healthcare facilities to primary care settings<br><br>Integration: Facility-based trainings and quality improvement steps for FP services in post abortion care	None	Post-intervention improvements in service indicators, including client waiting times, recall of emergency procedure counseling, contraceptive uptake, and satisfaction with the quality of overall counseling and FP information and services
Benson 2018, Senegal[36]	Evaluation study	Pre- and post-intervention data from women and health facilities	Women of reproductive age (15–49)	FP services: Community-level activities: (1) Training of religious leaders to become FP champions and conduct meetings in the community (2) mass media strategies including messages on radio, television and in print media (3) training of community-health volunteers to share information about FP through one-on-one or group discussions with women and other members of the household, and (4) community-level FP awareness activities<br><br>System-level activities: novel stocking and commodity tracking system, training providers; strengthening the referral system through training on the use of a systematic screening tool to identify client's FP needs; deploying midwives into facilities with gaps in services; and improving the private sector through pharmacy strengthening and	None	Modern contraceptive use increased from 16.9% to 22.1% with a slightly larger increase among the poor (16.6% to 24.1%)<br><br>Women exposed to community-based activities were more likely to use modern contraception by endline (marginal effect (ME): 5.12; 95% confidence interval (CI): 2.50–7.74) than those not exposed<br><br>Women living within 1 km of a facility with family planning guidelines were more likely to use (ME: 3.54; 95% CI: 1.88–5.20) than women without a nearby facility with guidelines

				support to Marie Stopes International in augmenting their FP services<br><br>MCH: Routine maternal and child health program by the government<br><br>Integration point: Integrating FP services (demand and supply sides) into Senegal Urban Reproductive Health Initiative		
Bhadra 2018, India[37]	Operations research	Service statistics	Women attending the prenatal clinic or labor room in early labor	FP services: FP counseling during prenatal visits and at the time of admission (before delivery but not when in active labor), (2) women who provided consent had an IUD inserted by either a doctor or a nurse within 10 minutes of placental expulsion in vaginal deliveries, (3) intraoperative insertion at cesarean delivery by doctors only, and (4) follow-up at 6 weeks after insertion<br><br>MCH services: Trained nurses provided regular health services including nutrition services during antenatal care visits.<br><br>Integration point: FP services provision by trained nurses during antenatal care and delivery	None	37.4% PPIUD acceptance rate<br><br>92.8% vaginal PPIUD insertions performed by trained nurses in the labor room<br><br>63.4% of women returned for follow-up and, of these, 93.7% reported a willingness to continue with the PPIUD
Biswas 2017, Bangladesh[38]	Evaluation study	Pre- and post-exist interviews with clients	Women attending health facilities	FP services: Key messages on importance of contraception no later than 8 weeks after birth; the location of the nearest family planning clinic; the choice of methods; the availability of sterilization services at the hospital<br><br>MCH services: Key messages included the advantages of breastfeeding, dangers of bottle feed, increasing milk supply, dangers and risks of diarrhea, symptoms and responses to respiratory infections and the importance of immunizations	None	Proportion of women accepting a postabortion contraceptive method increased from 14.3% at baseline to 69.2% at endline in Directorate General of Health Services facilities

				Integration point: Training of doctors and nurses in providing reproductive and sexual health services including FP		
Bolam 1998, Nepal[39]	Randomized controlled trial	Community follow up at 3 and 6 months postpartum by interview	Pregnant women	FP services: Two one-to-one health education sessions; the importance of exclusive breast feeding in the first session and on the need for family planning in the second session; first session in the hospital and second session at client's home. Sessions conducted by female health educators, midwives, and community health worker<br><br>These two interventions provided in three interventions arms: Arm I: health education given immediately after birth and 3 months later; Arm II: education given at birth only; Arm III: education given at 3 months only<br><br>MCH services: Routine MCH services; no further details provided<br><br>Integration Point: Integration of maternal and child health services and FP by provider at service point	No intervention	Mothers who received health education at birth were more likely to use contraception at six months after birth compared with mothers who received no health education at birth<br><br>No other significant differences between groups with regards to infant feeding, infant care, or immunization
Bongiovanni 2005, Jordan[40]	Evaluation study	Post-intervention survey with women and healthcare providers	Women who had a child 13-24 months of age	FP services: Promotion of breastfeeding and LAM through television and radio spots, posters, clinic-based brochures, and desk flipcharts<br><br>MCH: postpartum health and nutrition care to mothers and their child<br><br>Integration point: Training of ministry of Health primary health staff to strengthen the capacity to integrate breastfeeding counseling, lactation management and LAM into the service delivery system	None	One-third of respondents relied on breastfeeding as their first method of family planning in the six months following the birth of their child<br><br>Modern method use at 12 months was higher among LAM users (40.5%) compared to Breast Feeding for FP users (23.3%), to non-family planning users (13.7%), and to users of traditional methods (12.7%)

Brooke 2015, Zimbabwe[41]	Evaluation study	Quarterly client interviews and pregnancy testing during 12-month follow-up period	Women treated for incomplete abortion	FP services: Ward-based family planning services prior to discharge including information and counseling about short- and long-term fertility control and the option to receive condoms, oral contraceptives, or the injectable Depo Provera prior to leaving the hospital; referral for clients who desired implants, intrauterine devices, or other methods<br><br>Abortion care: Clinic-based treatment for incomplete abortion (no specific description)<br><br>Integration point: Two-week training of two gynecology nurses, four hospital-based distributors, and two research coordinators in the health facility	Treatment for incomplete abortion, but no provision of ward-based family planning service	During the follow-up period, significantly more women used highly effective methods of contraception, significantly fewer unplanned pregnancies occurred, and fewer repeat abortions were performed at the intervention site than at the control site
Camara 2018, Republic of Guinea[42]	Quasi experimental study	Interviews with women from sixth month of pregnancy and follow up until the ninth month postpartum	Pregnant women	FP service: In addition to routine antenatal group counseling session, a face-to-face individual counseling focused on postpartum FP methods (modern and traditional methods). FP methods were available in the selected health centers: pills, injectable methods, implant, intrauterine device (IUD), male and female condoms<br><br>MCH: nutrition, childbirth preparedness, immunization counseling during antenatal visits<br><br>Integration point: Antenatal care providers trained to provide specific counseling session	Routine antenatal group counseling covering childbirth preparedness, immunization and family planning	At the ninth month postpartum, use of modern FP was significantly higher in the intervention group than in the control group (5.7% and 1.1%, respectively)
Ceylan 2009, Turkey[43]	Evaluation study	Post-intervention follow-up interviews	Women who had induced abortion one year ago	FP services: Post-abortion counseling session and follow-up by telephone calls or by home visits to take information on their complains about the chosen contraceptive method.	None	Before induced abortion performed, the total contraception usage (modern and traditional methods) rate was 44.7% and this rate increased to 80.1% at the

				Abortion care: Clinic-based post abortion care (no specific details provided)<br>Integration point: 3 physicians and 7 nurses from a hospital were trained on adult training principles and counseling skills<br><br>Integration point: Abortion clinic staff trained for providing FP services		end of one year post training of the service providers<br><br>Modern method of contraception usage increased to 62%, with the major increaser being in the use of IUDs. No women prior to abortion had IUDs, whereas 52% were availing of IUD contraceptives 1 year later
Charurat 2010, Nigeria[44]	Evaluation study	Pre- and post-intervention observations of provider-client interactions; provider interviews; client exit interviews; and service statistics	Reproductive age women	FP services: (i) Providing LAM transition counseling for women who meet the three LAM criteria; (ii) Providing postnatal FP counseling for women who have a child underage of two and is not using any FP method; (iii) providing general FP counseling for women who do not want to get pregnant and is not using any FP methods<br><br>MCH services: Child immunization services within clinic<br><br>Integration point: Service providers in immunization, newborn care and pediatric/sick baby units trained in communication and counseling skills and using postpartum screening checklist	None	Women attending immunization, newborn care and pediatric/sick baby services were more likely to be screened for FP, postnatal care, and immunization services (17% vs. 68%, 13% vs. 57% and 47% and 89%, respectively)<br><br>73% of trained providers knew at least three family planning methods suitable for postpartum women, and all of them were providing family planning counseling to pregnant or postpartum women
Chin-Quee 2016, Rwanda[45]	Quasi experimental study	Longitudinal and cross-sectional data collected from CHWs and their clients	Pregnant and postpartum women	FP services: A pair of male and female CHWs in each village provided family planning counseling, referral, and condom provision. These CHWs also provided re-supply of oral contraceptive pills and DMPA.<br><br>Health services: CHWs provided community-based integrated management of child illness; growth monitoring of infants and children; directly observed treatment of tuberculosis; rapid tests for	Except the re-supply of contraceptives, the CHWs provided all the services as the intervention arm.	CHW diaries did not reveal significant differences between intervention and control groups in time spent on service provision or travel<br><br>Over 90 % of CHWs reported workload manageability, job satisfaction, and motivation to perform their jobs<br><br>Clients were highly satisfied with CHW services and most stated

				malaria diagnosis; nutrition counseling; counseling on sanitation and home hygiene; as well as HIV/AIDS prevention and support for people living with HIV/AIDS<br><br>Integration point: Training CHWs to provide family planning services along with regular duties		preference for future services from CHWs
Cooper 2013, Liberia[46]	Evaluation study	Mid- and end-line assessment using quantitative and qualitative methods; Service statistics; observations during supportive supervision visits and training activities; qualitative investigation	Postpartum women	FP services: (1) During routine infant immunization sessions, targeted FP and immunization messages one-on- one with mothers and referral to co-located FP room for more in-depth FP counseling and services, (2) women interested in seeking FP services on the same day directed to the FP room by the vaccinator, (3) women who are not interested in seeking FP on the same day are given a leaflet with information (4) Posters display in the clinic<br><br>MCH services: Child immunization by the vaccinators<br><br>Integration point: Integration of FP services in EPI clinics in the same facility	None	Large increases in the numbers of new contraceptive users in intervention clinics compared with pre-intervention service data<br><br>Pilot facilities experienced an increase in the number of doses of Penta 1 and Penta 3 administered<br><br>Activities increased vaccinators' sense of confidence and value within the India and community
Cooper 2014, Bangladesh[47]	Quasi experimental study	in-depth interviews with postpartum women and focus group discussions (FGDs) with mothers/ mothers-in-law and husbands of postpartum women	Pregnant and postpartum women	FP services: (1) Behavior change communication messages related to postpartum FP added to the existing MCH intervention package for interpersonal counseling and group meetings (2) oral contraceptives and condoms provided by community health workers, and (3) monthly meetings organized by community mobilizers for men and women to discuss the importance of pregnancy spacing and postpartum FP, including LAM<br><br>MCH services: Regular postpartum services to mothers and their child postpartum visits by CHWs	Maternal and newborn-care services only	Reported shifts in perceived susceptibility to pregnancy, benefits of pregnancy spacing, and increased social support for postpartum family planning (PPFP) were noted<br><br>Only approximately one third of women were using a modern contraceptive method

				Integration point: FP services linked with the facility by CHWs during postpartum visits		
Cooper 2015, Liberia[48]	Evaluation study	Baseline, midline and endpoint assessment and qualitative interviews and FGDs; and service data	Postpartum women	FP services: (1) During routine infant immunization sessions, targeted FP and immunization messages one-on- one with mothers and referral to co-located FP room for more in-depth FP counseling and services, (2) women interested in seeking FP services on the same day directed to the FP room by the vaccinator, (3) women who are not interested in seeking FP on the same day are given a leaflet with information (4) Posters display in the clinic<br><br>MCH services: Child immunization by the vaccinators<br><br>Integration point: Integration of FP services in EPI clinics in the same facility	None	Referral acceptance across the facilities varied from 10% to 45% per month, on average. Over 80% of referral acceptors completed the family planning visit the same day, of whom over 90% accepted a contraceptive method that day<br><br>Total number of new contraceptive users at participating facilities increased by 73% in Bong and by 90% in Lofa<br><br>Little difference in the number of Penta 1 and Penta 3 doses administered between pilot and non-pilot facilities
Cooper 2016, Egypt[49]	Quasi experimental study	Pre- and post-intervention household surveys	Pregnant and postpartum women	FP services: (1) CHWs reached out to pregnant and postpartum women to counsel them on a range of reproductive and MCH topics, including the benefits of family planning, healthy timing and spacing of pregnancies, postpartum return to fecundity and risk of pregnancy after childbirth, LAM, and timely transition to other modern contraceptive methods. (2) CHWs organized community meetings of men, mothers-in-law, and other community members. (3) Pharmacists encouraged to provide information on FP and discount on contraceptives (4) project facilitated mobile clinics for women<br><br>MCH services: maternal and child care home visits, nutrition classes and monthly seminars	Routine maternal health services by CHWs	In Upper Egypt, modern contraceptive use decreased over the study period in both intervention and comparison sites (by six and 15 percentage points, respectively), and in Lower Egypt, contraceptive use remained unchanged in intervention sites and decreased slightly (by three points) in comparison sites

				Integration point: CHWs trained to provide FP services in addition to MCH services		
Coulibaly 2021, Burkina Faso & Democratic Republic of the Congo[50]	Clustered randomized controlled trial	Secondary analysis of follow-up data up to 12 months after delivery	Pregnant women	FP services: (1) PPFP refresher course for providers, a counseling tool, supportive supervision, and availability of contraceptive services seven days a week (2) invitation letter to husbands or partners, appointment card, and a decision-making tool for clients and providers to enable a systematic counseling approach on PPFP<br><br>MCH services: Routine antenatal and postnatal care<br><br>Integration point: Clinic-based providers trained to provide FP counseling and commodities	Routine antenatal and postnatal care	An increase in family planning use in these two African countries<br><br>Yam Daabo's interventions showed a reduction of the median time of long-acting reversible contraceptives adoption in the intervention group compared to the control group in both countries (difference of 39 days in Burkina Faso; difference of 86 days in the DR Congo)
Coulibaly 2021, Burkina Faso[51]	Clustered randomized controlled trial	Follow-up of women to 12 months postintervention	Pregnant women	FP services: (1) Postnatal FP refresher course for providers, a counseling tool, supportive supervision, and availability of contraceptive services seven days a week (2) invitation letter to husbands or partners, appointment card, and a decision-making tool for clients and providers to enable a systematic counseling approach on postpartum FP<br><br>MCH services: Routine antenatal and postnatal care<br><br>Integration point: Clinic-based providers trained to provide FP counseling and commodities	Routine antenatal and postnatal care	No difference was observed in the use of hormonal contraceptive methods between the intervention and control groups<br><br>Women in the intervention group were more likely to use long-acting reversible contraceptives than those in the control group
Davis 2009, Burundi[52]	Operation research	Service data	Reproductive age women and their under five children	FP services: Two strategies to integrate family planning in community-integrated management of childhood illness. At the community level, it used its existing community mobilization platforms as the	None	Despite the short implementation period of 9 months, the program was able to increase the use of modern family planning methods from 16% at baseline to 42.7% at final evaluation

				entry point to deliver birth spacing interventions. At the health center level, two providers per facility were trained; (1) primarily nurses trained in family planning counseling and service delivery of modern and natural methods and, (2). CHWs referred women to health centers for family planning services and worked with health center staff to follow up with dropouts<br><br>MCH: Community-integrated management of childhood illness including nutrition<br><br>Integration point: Linkages between existing community mobilization platform and health centers through training to CHWs and nurses		
Delvaux 2008, Cambodia[53]	Operations research	Routinely collected data in clients' medical files	Women who availed safe abortion ⁄ post-abortion clinic services	FP services: Family planning counseling<br><br>Abortion care: Management of sexually, and the manual vacuum aspiration procedure for first trimester abortion and post-abortion services. Strict sterilization and disinfection procedures were put in place<br><br>Integration point: MCH clinic staff trained on safe abortion⁄ post-abortion clinics	None	Between January 2003 and the end of 2005, 40% (792 ⁄ 1970) of SAPAC clients adopted a modern contraceptive method after the abortion<br><br>Of women using post-abortion contraception, 34% used the pill, 32% opted for an IUD, 27% for injectables and 7% for condoms
Dhital 2020, Nepal[54]	Evaluation study	Mixed methods study using knowledge assessment of service providers, coverage data, and qualitative investigation with users and providers	Pregnant and postpartum women	FP services: Female community health volunteers (FCHVs) conduct mothers' group meetings in the communities every month, organize counseling sessions for pregnant women in the communities on FP, birth preparedness and the advantages of institutional delivery. FCHVs identify danger signs among pregnant and post-partum mothers and newborns through their antenatal and postnatal home visits and	None	FCHVs' knowledge and community-based activities on postpartum family planning remained higher than in the pre-intervention<br><br>Postpartum mothers in hospitals had 2 times higher odds of being counseled by FCHVs during their pregnancy at one-year post-intervention than in pre-intervention phase

				refer any with complications to health facilities<br><br>MCH: regular postpartum care to mothers and their child through FCHVs through home visits<br><br>Integration point: Training of FCHVs to integrated FP in the existing maternal and newborn health program		
Dimond-Smith 2020, India[55]	Evaluation study	Mixed-methods evaluation using surveys with women seen by ASHAs that did and did not receive this additional training and qualitative interviews with ASHAs who received the intervention.	Pregnant and postpartum women	FP services: Addition to a standard in-service family planning training. Accredited social health activists (ASHAs) received an additional training module focused on salient person-centered FP domains. Training covered: the importance of person-centered care, FP method mix and supporting women in choosing appropriate FP methods for themselves.<br><br>MCH services: Routine services including nutritional counseling by ASHAs<br><br>Integration point: Community health workers cover a range of maternal and child health programs and FP integrated with it.	Standard in-service family planning training to ASHAs	Comparing women who saw intervention compared to control ASHAs, the intervention had no impact on overall person-centered family planning scores.<br><br>Women in the intervention arm were more likely to report that their ASHA had a strong preference about what method they choose, suggesting that the training increased provider pressure
Douthwaite 2005, Pakistan[56]	Evaluation study	Quantitative and qualitative data collection, a review of Program management systems and an analysis of Program expenditure	Pregnant and postpartum women	FP services: Lady health workers (LHWs) promote family planning including motivating women to practice family planning, providing pills and condoms, and referring for injections, IUD and sterilization<br><br>MCH services: LHWs promote childhood immunization, growth monitoring, health promotion and education, and treat minor ailments and injuries, and refer more serious cases to health facility	Households not served by the LHWs	Women served by Lady Health Workers are significantly more likely to use a modern reversible method than women in communities not served by the Program (OR 1.50, 95% CI 1.04–2.16, p= 0.031)

				Integration point: FP-MCH services integrated for delivery by frontline health workers		
Dulli 2016, Rwanda[57]	Clustered randomized controlled trial	Pre- and post-intervention implementation assessments and service data	Postpartum women attending immunization services for their infant	FP services: (1) Concise messages delivered during group education sessions, (2) distribution of a simple brochure during group session, (3) individual screening of all women attending infant immunization services by the immunization provider, and (4) Convenient offer of family planning services to women attending immunization at the same facility and on the same day as immunization service<br><br>MCH services: Immunization for infants of postpartum women<br><br>Integration point: Integrated family planning and immunization services at the same time and location	Standard immunization services only	Intervention had a statistically significant, positive effect on modern contraceptive method use among intervention group participants compared with control group participants (regression coefficient, 0.15; 90% CI 0.04 to 0.26)<br><br>Women in both study groups overwhelmingly supported the concept of integrating family planning service components into infant immunization services (97.9% in each group)
Erhardt-Ohren 2020, Benin[58]	Process evaluation	FGDs with postpartum women who attended integrated sessions	Postpartum women	FP services: (1) Brief, targeted messages at FP group education sessions about the benefits and availability of FP services, (2) a facilitator offered the women referral cards for individual counseling with nurse midwives, and (3) contraceptive method of choice on the same day and at the same facility or later when needed<br><br>MCH services: Immunization for infants of postpartum women<br><br>Integration point: Integrated family planning and immunization services at the same time and location	None	Contraceptive use was motivated mainly by a desire for birth spacing, whereas fear of side effects and lack of spousal engagement were cited as reasons for contraceptive non-use<br><br>Women cited the referral process as confusing
Fatima 2018, Bangladesh[59]	Operations research	Service data collected five months before and five months after the training	Postpartum women	FP services: (1) Training of healthcare providers in postpartum FP counselling and PPIUD insertion, enabling them to be incorporated into routine prenatal counselling and delivery room practice and,	None	Increase in the proportion of women who were counselled (from 75.3% to 83.8%, P<0.001) pre- and post-training of counselors

				(2) Training of counselors to provide individual face-to- face and group counselling during the prenatal period, in early stages of labor, and within the first 48 hours of delivery in outpatient department<br><br>MCH services: Community-based services to postpartum women in the community through lay counselors<br><br>Integration point: Provision of FP services in the healthcare facility during antenatal, early stages of labor and early postpartum period		Small decrease in the proportion of women agreeing to have a PPIUD inserted following counselling (13.7% vs 12.9%, P=0.03) from pre- and post-training of counselors
FHI 360, Rwanda[60]	Clustered randomized controlled trial	Pre- and post-intervention assessments with structured observations of immunization services and supervisory data	Postpartum women and their infants	FP Services: (1) Health providers delivered short messages to women attending routine infant immunization services during group education sessions, (2) distributed educational brochures, (3) used a screening tool with all mothers to assess risk of an unplanned pregnancy, using criteria based on LAM and, (4) a brief counseling message depending upon a mother's risk classification, including a referral for same-day FP services<br><br>MCH services: Immunization for infants of postpartum women<br><br>Integration point: Integrated family planning and immunization services at the same time and location	Standard immunization services only	At baseline, contraceptive prevalence in the intervention sites was 49% and increased to 57% prevalence at follow-up. In the control group, prevalence at baseline was 58% and declined to 51% at follow-up
Fotso 2015, India[61]	Qualitative evaluation	Interviews with frontline workers, clients, and their husbands	Women and their children	FP services: Male health workers trained to encourage men to take a more active role in the health of mothers and children including antenatal care, immunization, growth monitoring of children under the age of five years, FP counseling to men, distribution of contraceptives, provision of drugs to	None	Complementarity of male and female community health workers in the community-based delivery of, and increased demand for maternal, neonatal, child health services

				patients as required, and supplementary nutrition to pregnant women<br><br>MCH services: Coordination of outreach and community based rural MCH services<br><br>Integration point: Strengthening of the existing maternal, neonatal, child health program delivered by frontline female workers		
Gage 2023, Democratic Republic of the Congo[62]	Quasi experimental study	Baseline and 16 months follow-up interviews	First-time mothers aged 15-24	FP Services: Trained nursing students provided (1) monthly group education sessions and home visits that included client-centered counseling on postpartum FP and birth spacing, (2) offered a range of contraceptive methods, and (3) provided referrals.<br><br>MCH services: Group education sessions on equitable gender roles, empowerment in interpersonal relationships, and sexual and reproductive health and rights to first time mothers in the community<br><br>Integration point: Integration of FP and MCH services	Standard maternal and newborn health services	Intervention group had a significantly higher probability of receiving FP counseling from a community-based health worker, obtaining the current contraceptive method from a community-based health worker, informed choice, and of currently using implants vs. other modern methods than the comparison group
Gallo 2013, Madagascar[63]	Evaluation study	Cross-sectional interviews with community health workers	Reproductive age women	FP services: CHWs were trained to provide basic family planning services including counselling and provision of short-acting methods (condoms, oral contraceptive pills and injectables) and referrals for information on and access to long-acting reversible contraception (LARC) and permanent contraceptive methods<br><br>MCH services: CHWs were trained in early detection of pregnancy and referral of patients to antenatal care	None	CHWs proved capable of providing high-quality contraception services; more education, more weekly volunteer hours, and receiving a refresher training correlated with a higher performance

				Nutrition services: CHWs were trained in nutrition counselling and provision of iron and folic acid tablets<br><br>Integration point: maternal, reproductive health and family planning services through CHWs		
Guo 2022, Nepal[64]	Stepped-wedge cluster randomized trial	Survey data on women immediately after delivery and at 1- and 2-years postpartum follow-ups	Postpartum women	FP services: (1) Training of providers at the hospital and associated clinics for in-person PPIUD-specific and general family planning counseling, (2) distribution of PPFP leaflets to women during ANC, (3) an information wall chart and a video broadcast in the antenatal care waiting room on FP and PPIUD, and (4) training and supplies for PPIUD insertion/removal and techniques and complication management.<br><br>MCH services: Hospital staff provided routine MCH services<br><br>Integration point: PPIUD training to hospital staff	No intervention during waiting period	Adjusted probability of having incident pregnancy was 0.7 percentage points (95% CI -3.0, 1.4) lower among women in the intervention group than among women in the control group<br><br>Women in the intervention group more likely to receive antenatal FP counseling and to receive antenatal PPIUD counseling than women in the control group
Hackett 2020, Tanzania[65]	Qualitative evaluation	Interviews with providers and women participating in the initiative	Postpartum women	FP services: (1) Trained maternity care providers at tertiary/teaching hospitals on PPFP counseling, PPIUD insertion techniques, and complications management; (2) informational workshops for nurses and midwives at satellite clinics on PPFP counseling techniques; (3) PPFP leaflets to be distributed during counseling; (4) a video to be played in hospital waiting areas; (5) supplied Kelly forceps for vaginal PPIUD insertion; and (6) conducted regular monitoring and support<br><br>MCH services: Hospital staff provided routine MCH services	No intervention during waiting period	Health system and resource constraints made adoption and fidelity to the intended intervention challenging<br><br>Providers questioned the sustainability of the initiative, and most agreed that changes to the initiative's design (e.g., additional training opportunities, improved staffing, and availability. of PPIUD supplies) would strengthen future iterations of the initiative<br><br>Interpersonal aspects of care varied, with some women reporting rushed or incomplete counseling or an emphasis on the PPIUD over other methods

				Integration point: PPIUD training to hospital staff		
Hamon 2020, Malawi[66]	Qualitative evaluation	Semi-structured interviews and FGDs with stakeholders	Postpartum women and their children	FP services: (1) Group information session on FP, (2) individual counseling for modern methods of contraception, (3) provision of pills, injectables and condoms in the same location, and (4) referral for permanent method) or long-acting reversible contraceptive to nearest health facility<br><br>MCH services: Immunization for infants of postpartum women during outreach clinic visits<br><br>Integration point: FP and childhood immunization services at monthly routine outreach clinics	None	Women were motivated to attend outreach clinics due to shorter travel distances; they felt confident they could access FP services and use contraceptive methods covertly if needed<br><br>Providers were empowered through the training they received to provide integrated services; they were confident in their ability to provide essential services
Hamon 2022a, Malawi[67]	Qualitative evaluation	Interviews with clients and providers and clinic audit	Postpartum women and their children	FP and immunization services: (1) Group information session on FP, (2) individual counseling for modern methods of contraception, (3) provision of pills, injectables and condoms in the same location, and (4) referral for permanent method) or long-acting reversible contraceptive to nearest health facility<br><br>MCH services: Immunization for infants of postpartum women during outreach clinic visits<br><br>Integration point: FP and childhood immunization services at monthly routine outreach clinics	None	Overall, 13 factors were found to influence service responsiveness in terms of the ease of access, choice of provider, environment, service continuity, confidentiality, communication, dignity and FP counselling afforded to clients
Hamon 2022b, Malawi[68]	Qualitative evaluation	Exit interviews with clients and clinic audits	Postpartum women and their children	FP and immunization services: (1) Group information session on FP, (2) individual counseling for modern methods of contraception, (3) provision of pills, injectables and condoms in the same location, and (4) referral for permanent	None	FP services can be responsive when integrated with childhood immunizations, particularly in terms of the dignity and service continuity afforded to clients, though less so in terms of the choice of provider,

				method) or long-acting reversible contraceptive to nearest health facility<br><br>MCH services: Immunization for infants of postpartum women during outreach clinic visits<br><br>Integration point: FP and childhood immunization services at monthly routine outreach clinics		environment, and confidentiality experienced
Harris-Fry, 2016, Bangladesh[69]	Cluster randomized trial	Pre- and post-intervention interviews with women	Reproductive age women	FP services: Identifying strategies to address the problems including family planning, nutrition and sexual health issues and collectively planning, implementing and evaluating<br><br>MCH services: Maternal and neonatal health, and focused on pregnancy, delivery and postnatal health risks for women and babies<br><br>Integration point: Integrated FP-MCH services in women's group	Routine care	Significant improvements in women's dietary diversity score and participation in healthcare decision-making. There were also increases in knowledge about contraception, ways to treat and prevent sexually transmitted infections, nutrition and anemia
Harrison 2020, Guatemala[70]	Clustered randomized trial	Data collected at 3- and 12-months follow-up visits	Postpartum women	FP services: along with routine care, nurses offered contraceptives (condoms, pills, syringe of medroxyprogesterone and implant), follow-up care and referral to nearby facilities<br><br>MCH services: Routine postpartum care<br><br>Integration point: FP integration in routine postpartum care	Routine care including prenatal contraceptive counseling and education	Three-month contraceptive initiation rates were 56.0% in the control clusters compared to 76.8% in the intervention clusters<br><br>Implant uptake was 25% in the intervention cohort compared to 3% in the control clusters<br><br>Women in control clusters overwhelmingly opted for the injectable contraceptive (94.6%) while women in intervention clusters chose both the injection (61.5%) and the implant (33.7%)

Harrison 2021, Guatemala[71]	Clustered randomized trial	Nurse survey and FGDs, and a patient survey	Postpartum women	FP services: Along with routine care, nurses offered contraceptives (condoms, pills, syringe of medroxyprogesterone and implant, follow-up care and referral to nearby facilities<br><br>MCH services: Routine postpartum care<br><br>Integration point: FP integration in routine postpartum care	Routine care including prenatal contraceptive counseling and education	Intervention achieved good reach (89% of the eligible population) and was acceptable to the majority of patients and providers
Hemono 2022, Rwanda[72]	Evaluation study	Cross-sectional survey with youth in schools and interviews (IDIs) with program stakeholders	12-19 years old students	Sexual and reproductive health program with components on contraception: (1) web-based fun and comic content, (2) web-based library of Q&As and directory of health facilities, and (3) discrete and private shopping of contraceptives and other materials<br><br>Integration point: FP/SRH integration into comprehensive sexuality education	None	High demand and enthusiasm for the CyberRwanda program among students<br><br>High acceptability of FP/RH content but few online purchases of contraceptives
Hersh 2018, Colombia[73]	Randomized controlled trial	Pre- and post-intervention assessment and process data	Prenatal or women entering labor	FP services: (1) a short video providing information on FP, (2) Conversation with a counselor, and (3) provision of the modern method mix. Women also received written material<br><br>MCH services: Routine maternal care<br><br>Integration point: Integration of FP services in maternal care	A structured conversation with trained counselor; content was similar to the video intervention	Compared to in-person contraceptive counseling alone, video-based intrapartum contraceptive education took a similar amount of time and resulted in similar contraceptive knowledge acquisition, though with fewer patients choosing LARC
Hodges 2015, Sierra Leone[74]	Process evaluation	Service data	Postpartum women and their children	FP services: (1) FP counseling by trained nurse aid in the clinics, (2) informed decision making, (3) provision of chosen method, (4) referral for a long-term method, and (follow-up visits<br><br>MCH services: immunization and vitamin A supplementation for infants of postpartum women	Vitamin A supplementation for infants and children and/or infant and child feeding counseling and demonstration and health card	FP commodities were provided to 44.5 % of caregivers in the full compared with 2.5 % in the vitamin A supplementation and new child health card only groups

				Integration point: FP services during vitamin A supplementation and immunization visit for infants and children		
Hoyt 2021, Benin, Ethiopia, Kenya, Malawi, & Uganda[75]	Qualitative evaluation	Interviews and FGDs with women, health providers, and community members	Women and their children	FP services: Integration model varied by country and site; but broadly the intervention included co-locating, to varying degrees, messaging, counselling and the provision of modern contraceptive methods<br><br>MCH services: Immunization and vitamin A supplementation for infants of postpartum women<br><br>Integration point: FP and childhood immunization services at co-location clinics	None	Integrated FP and childhood immunization services expanded women's choices about modern contraceptive methods use and created opportunities for women to make decisions autonomously
Huang 2014, China[76]	Evaluation study	Post-intervention follow-up data	Pregnant women	FP services: (1) Couple counseling session on FP and pamphlet distribution, (2) accessibility of contraceptives, (3) long-acting contraceptive methods were provided prior to hospital discharge, and (4) investigators number for contacting for MCH and FP related problems in the future<br><br>MCH services: Routine maternal care<br><br>Integration point: Clinic-based child-birth care and FP services	None	Among all study participants, the median time to contraceptive initiation and sexual resumption was 2 months postpartum, respectively<br><br>Overall contraceptive prevalence at 12 months was 97.1%, and more than half of the women were using long-acting contraception
Huber 2010, Afghanistan[77]	Evaluation study	Pre- and post-intervention surveys	Women and children	FP services: Volunteer CHWs provided health care for women and children, including providing information about the safety and non-harmful side-effects of contraceptives and improving access to injectable contraceptives, pills and condoms<br><br>Community-health services: CHWs provided routine health services to women and their children; no further details provided	None	Contraceptive prevalence rate increased by 24–27% in 8 months in the project areas. Men supported modern contraceptives once they understood contraceptive safety, effectiveness and non-harmful side-effects. Injectable contraceptives contributed most to increases in contraceptive use

				Integration point: FP services included in the community-based health care project		
Huber-Krum 2020, Nepal[78]	Stepped wedge randomized controlled trial	Survey data on women immediately after delivery and at 1- and 2-years postpartum follow-ups	Postpartum women	FP services: (1) Training of providers at the hospital and associated clinics for in-person PPIUD-specific and general family planning counseling, (2) distribution of FP leaflets to women during antenatal care, (3) an information wall chart and a video broadcast in antenatal care waiting room on PPFP and PPIUD, and (4) training and supplies for PPIUD insertion/removal and techniques and complication management<br><br>MCH services: hospital staff provided routine MCH services<br><br>Integration point: PPIUD training to hospital staff	No intervention during waiting period	Intervention increased use of modern contraceptives by 3.8 percentage points (95% CI: 0.1, 9.5) at one-year postpartum, but only 0.3 percentage points (95% CI: 3.7, 4.1) at two years<br><br>Intervention significantly increased the use of PPIUDs at one year and two years postpartum, but there was less use of sterilization
Jacinto 2016, Mozambique[79]	Evaluation study	Assessments with women at enrollment (first DMPA injection), at a follow-up visit after 3 months (13 weeks, second DMPA injection), and 1 at a follow-up visit after 6 months (26 weeks, third DMPA injection).	Reproductive age women	FP services: CHWs and traditional birth attendants (1) offer a new package of services that includes provision of pills, condoms, and injectables to women during labor, delivery, and the postpartum period in the community and in the health facilities<br><br>Community-based services: CHWs serve as a linkage between communities and health facilities, and they provide community case management for HIV/AIDS, maternal care, nutrition, and acute illness among children.<br><br>Integration point: Provision of integrated routine MCH and FP services by CHWs	None	Majority (63% to 66%) of women in the study started using contraception for the first time during the study period, and most women (over 66%) did not report side effects at the 3-month and 6-month follow-up visits<br><br>Satisfaction with the performance of TBAs and APEs was high and improved over the study period
Jackson 2016, Ghana[80]	Experimental evaluation study	Navrango Demographic Surveillance Data	Women aged 15–49 years	FP services; (1) community mobilization and male involvement by community health volunteers; and (2) doorstep family planning services offered by community health nurses using motorbikes	Regular maternal and child health services at the health facilities	Total fertility rate declined steadily from 1994 to 2003 in areas of community health and family planning project service areas<br><br>Over the same decade, the use of modern contraception increased by

				MCH services: Routine MCH services through community nurses and volunteers in community clinics<br><br>Integration point: Provision of integrated routine MCH and FP services		approximately 6 percentage points, and the median duration of postpartum abstinence decreased by roughly 5 months
Jarvis 2018, Democratic Republic of the Congo[81]	Evaluation study	Client exit-interviews conducted post-intervention	Women attending ANC, PNC, and immunization units	FP services: Quality inputs intervention including: (1) clinical training and provision of equipment for PPIUD, (2) training on the WHO's medical eligibility criteria for contraceptive use, and (3) introduction of a systematic screening and referral tool for family planning<br><br>Free methods arm: All contraceptive provided free of costs<br><br>Method availability arm: Clients could access IUDs, implants, injectables, oral contraceptives, and male condoms in the family planning department throughout the duration of the intervention<br><br>MCH services: Routine antenatal postpartum care and immunization services<br><br>Integration point: FP services delivered during routine MCH care	Routine care	Providing family planning, including LARCs, in the immediate postpartum period, implementing a systematic screening and referral system, and providing free methods improved family planning access and uptake in the extended perinatal period in this environment
Karra 2019, Sri Lanka[82]	Cluster -randomized stepped-wedge trial	Baseline and post-intervention surveys with women	Women who delivered in the selected hospitals	FP services: (1) workshops on PPFP and PPIUD for doctors, midwives, nurses, and general hospital staff who worked in maternity wards; (2) training of maternity care providers in hospitals and in s Ministry of Health on FP counseling; (3) training of doctors in study hospitals in PPIUD insertion; (4) provision of PPFP leaflets during counseling; (5) provision of a video in the hospital waiting area; (6) provision of Kelley's forceps for vaginal PPIUD insertion and of copper-T IUDs to hospitals;	No intervention during waiting period	Intervention increased rates of counseling, from an average counseling rate of 12% in all hospitals prior to the intervention to an average rate of 51% in all hospitals after the rollout of the intervention

				and (7) monitoring and evaluation of counseling activities and PPIUD insertions<br><br>MCH services: Hospital staff provided routine MCH services<br><br>Integration point: PPIUD training to hospital staff		
Kibel 2022, Kenya[83]	Qualitative evaluation	Pre-intervention FGDs with women, men, and CHVs, and post-intervention FGDs with community health volunteers	Women of reproductive age	FP services: Training of community health volunteers to deliver urine pregnancy testing, post-test counselling, and referral to care to support timely initiation of antenatal care and uptake of FP<br><br>MCH services: Antenatal care and other reproductive health services by health volunteers in the community<br><br>Integration point: Training of health volunteers to provide antenatal care and FP services	None	Training CHVs in the study context to deliver UPT, post-test counselling, and referral to care was acceptable and feasible to men, women, and CHVs, and promoted early initiation of ANC and uptake of FP
Kimani-Murage 2017, Kenya[84]	Cluster - randomized controlled trial	Every 2-months data collection on breastfeeding data through interviews	Pregnant women	FP services: Home-based counselling by CHWs on FP<br><br>MCH services: maternal and child care services by health professionals; personalized home-based nutritional counselling of women from the time of recruitment until the baby attained one year through scheduled visits at pregnancy - monthly until week 34, then weekly until delivery; mother and baby pairs – weekly in the first month then monthly until 12 months<br><br>Integration point: Home-based MCH and FP delivered by CHWs	Maternal, infant and young child nutrition routine practices through community health workers	The rates of exclusive breastfeeding for 6 months increased from 2% pre-intervention to 55.2% (95% CI 50.4, 59.9) in the intervention group and 54.6% (95% CI 50.0, 59.1) in the control group
Klinger 2016, Madagascar[85]	Evaluation study	Pre- and post-intervention surveys	Adolescents aged 15-19 years	Intervention components: (1) a comprehensive six session curriculum on HIV/STIs and family planning, (2)	None	Post-curriculum, there was a significant improvement in the following educational domains: general

				provision of supportive education material during sessions, and (3) anonymous question box to submit and address concerns and questions<br><br>Integration point: Teachers provided comprehensive sexuality education		knowledge of HIV/AIDS, other STIs and family planning, self-efficacy and use of contraceptives, and the overall combined scores of knowledge, attitude and self-efficacy
Komasawa 2019, Jordan[86]	Evaluation study	Pre- and post-intervention surveys	Women of reproductive age	FP services: Village Health Centers (VHC) activities: (i) providing a series of FP training for nurses or midwives; (ii) conducting workshops for doctors and midwives who were periodically serving at VHCs; (iii) providing basic medical equipment; furniture; and information, education, and communication materials required in FP services; (iv) conducting supervisory visits by maternal and child health supervisors and, (v) updating the FP service manual for VHC staff<br><br>Community-based activities: (i) supporting the establishment of a community health committee in each village; (ii) providing workshops to the committee members; (iii) encouraging committee members to make an action plan for the activities; (iv) monitoring their activities monthly; and (v) providing seed money for the first 4 months<br><br>MCH services: Village health center provided routine MCH services<br><br>Integration point: FP services integrated in the clinic- and community-based MCH services	No intervention	Compared with the control villages, significant effects observed in the intervention villages for the following domains: increasing the use of FP services at VHCs, participation in health promotion activities, and changing the sources of reproductive health information
Koroma 2019, Sierra Leone[87]	Evaluation study	Interviews with service in-charges at health facility and mothers	Mothers of children 6-23 months of age	FP services: Confidential quality family counseling, and provision of modern commodities by health workers	None	In communities served by the project, awareness and uptake of modern contraception exceeded national targets despite weak supply chains, and

				MCH services: Vitamin A supplementation to children aged 6-7 months and Infant and Child Feeding Counselling and Demonstration by health workers<br><br>Integration point Integrating reproductive and child health services and FP in the health facilities		complementary feeding practices were favorable compared with the national survey
Krishnaratne 2021, Ban[88]	Qualitative evaluation	Semi-structured interviews with project stakeholders	Postpartum women	FP services: 1) training and mentoring of facility-based health extension workers (HEW) on FP counselling and short-acting modern contraceptive methods (MCMs) provision and implant insertions; 2) expanding the range of MCM options available at health posts to include implant insertions (but not removals); 3) designing a job aid to support HEWs to improve immunization defaulter tracing; and 4) developing community engagement strategies that involved community leaders and kebele command posts<br><br>MCH services: First round of immunization to children after 6 weeks after birth in health posts<br><br>Integration point: Integration of child immunization and FP services at household visit or at a health or outreach post	None	Linking context and intervention components to the mechanisms they triggered helped explain the intervention outcomes, and more broadly how and for whom the intervention worked<br><br>Linking empirical mechanisms to constructs of implementation related theoretical frameworks provided a level of abstraction through which findings could be cumulated across time, space, and conditions by theorizing middle-range mechanisms
Levy 2021, Kenya[89]	Evaluation study	Baseline and endline surveys with adolescent girls and routine program data	Pregnant adolescent girls, adolescent mothers and their infants	FP services: No details provided<br><br>MCH services: Bi-monthly household visits by three cadres of health providers (i.e., mentors who were young mothers aged 19-30, male and female household facilitators, and supervisors) provided ANC, PNC, HIV and related services to HIV-vulnerable and HIV-positive adolescent girls, and the babies of adolescent girls for necessary health and social services	None	Uptake of modern family planning increased from 39% at baseline to 64% at end line ($P < 0.001$)

				Integration point: FP services included into routine MCH services		
Limbila 2013, Kenya[90]	Evaluation study	Baseline and endline surveys	Postpartum women	FP services: Community midwives trained (1) to provide contraceptive commodities (implants and IUDs), and (2) to refer clients, especially those with complications or whose needs could not be adequately addressed at the community level<br><br>MCH services: Midwives delivered routine maternal health services to women's home<br><br>Integration point: Expanding community midwives mandate to go beyond the provision of delivery services	None	The expanded community midwifery model improved clients' access to a comprehensive package of family planning, reproductive health and HIV services at the community level. However, the intervention was less successful in improving the provision of a continuum of care by community midwives
Lori 2018, Ghana[91]	Evaluation study	Immediate postpartum face-to-face or via cellphone survey interviews	Women registering for antenatal care	FP services: Educational content was delivered through picture cards, role-play, storytelling, and teach-back to small groups of women with similar gestational age. One complete ANC visit dedicated to family planning and exclusive breastfeeding as a LAM for birth spacing in both study groups.<br><br>MCH services: Focused antenatal care in group setting in health facilities through nurses<br><br>Integration point: FP services integrated into antenatal care	Standard individual antenatal care	Women who participated in group ANC were more likely to use modern and non-modern contraception than those in individual care. Those who participated in group ANC were more likely to exclusively breastfeed for more than 6 months than those in individual care
McPherson 2010, Nepal[92]	Process evaluation	Semi-structured interviews with project stakeholders	Pregnant women and their family members	FP services: education through booklet cards on use of modern family planning method to space or limit births to women of reproductive age<br><br>MCH services: Albendazole and iron supplementation during pregnancy, postpartum care of mother including examination, rest and nutritious food for mother	None	Booklet cards on danger signs and nutritious foods are particularly well-received. Cards on family planning and certain aspects of birth preparedness generate less interest. Husbands and mothers-in-law control decision-making for maternal and newborn care-seeking and related household-level behaviors

				Integration point: Extension of role of CHVs under Nepal Family Health Program		
Mochache 2018, Kenya[93]	Evaluation study	District Health Information System	Women of reproductive age	FP services: CHVs organized regular sessions with communities using an informational picture booklet focusing on promotion of FP uptake and utilization.<br><br>MCH services: Antenatal care and facility-based deliveries<br><br>Integration point: FP services integrated into MCH services using community-participatory approaches	None	A total of 570 Dialogue Model sessions were held in 12 community units associated with 10 intervention facilities<br><br>Facilities reported 15, 2 and 74% increase in uptake of family planning, antenatal care and facility-based deliveries, respectively
More 2017, India[94]	Clustered randomized controlled trial	Census interviews at before and 2 years after the intervention	Women aged 15–49 years	FP services: Community organizers provided family planning directly (condoms and oral contraceptive pills) or by referral under a formal memorandum; family planning communication through adolescent sexual and reproductive health training<br><br>MCH services: Community organizers in the intervention centers addressed maternal and neonatal health, child health and nutrition, reproductive health, and prevention of violence against women and children through home visits, group meetings, day care, community events, service provision, and liaison<br><br>Integration point: FP services integrated into MCH services	Care as usual	Met need for family planning was greater in the intervention clusters than in the control clusters<br><br>Proportions of fully immunized children were similar in the intervention and control groups in the intention-to-treat analysis<br><br>Childhood wasting did not diff er between groups
Mwakangalu 2018, Kenya[95]	Program report	Program data	Reproductive age women, mothers, and their children	FP services: Health workers both at the facility and community levels trained and sensitized on FP counseling, information, method provision, and referral<br><br>MCH services: Health workers trained to provide information on exclusive breastfeeding education, and vitamin A	None	High acceptance of the services among clients<br><br>High sense of attention among clients from health workers

				supplementation, deworming, counselling, and support for children under five<br><br>Integration point: During outreach services, information and services on FP/RH are provided as part of the integrated package of care		Innovative outreach models like services under one roof and camel outreach were feasible
Mwakangalu 2020, Kenya[96]	Program report	Program reports using assessments, exit interviews with clients and service providers and service data	Reproductive age women, mothers, and their children	FP services: Health workers both at the facility and community levels trained and sensitized on FP counseling, information, method provision, and referral<br><br>MCH services: Health workers trained to provide information on exclusive breastfeeding education, and vitamin A supplementation, deworming, counselling and support for children under five<br><br>Integration point: During outreach services, information and services on FP/RH are provided as part of the integrated package of care	None	Integration of FP and reproductive health into program activities contributed to reduced waiting time for clients<br><br>High workload, limited infrastructure and equipment for maintaining privacy and confidentiality posed challenges to integration
Nelson 2019, Liberia[97]	Evaluation study	Service data and qualitative investigation	Mothers and their children	FP services: Family planning providers reviewed the child health cards of postpartum women who had come with their infant to the health facility primarily for family planning services, if available, and provided reminders about their child's next vaccination date. If the date had already passed, they referred the woman and child to the vaccinator for same-day immunization services<br><br>MCH services: Vaccinators trained to share brief family planning messages and refer postpartum women to same-day co-located family planning services	None	Trends indicated slightly higher family planning uptake in intervention over nonintervention facilities, but differences were not statistically significant<br><br>Clients and providers expressed that the integrated services reduced costs and time for the clients, educated mothers about postpartum family planning, and ensured infants were completing their vaccinations.<br><br>Providers expressed the need for increased human resources to meet the elevated demand for family planning counseling services and additional

				Integration point: Integrated FP and immunization services with staff training and referral		focus on community-level social and behavior change activities
Özçelik 2020, Pakistan[98]	Evaluation study	Data collected as part of large-scale community-based reproductive health initiative	Married women of reproductive age	FP services: Field educators conducted two visits i.e., counseling, and drop-in visits; counseling visits provided information on available contraceptive methods, prenatal, postpartum care, and postpartum contraception. Field educators referred women to health care providers and coordinated with the health care providers. During drop-in visits, field educators inquired about women's satisfaction with services to ensured sustained used of contraceptives and other services<br><br>MCH services: Field investigators provided information and referral services on reproductive health care and health rights through visits to married women of reproductive age<br><br>Integration point: Integrated MCH and FP services	None	Estimates made using inverse probability weighting methods suggested that the contraceptive prevalence rate increased from 51% to 64%, and the modern contraceptive prevalence rate increased from 34% to 53%, during the study
Palinggi 2021, Indonesia[99]	Quasi-experimental study	Pre- and post-intervention assessments	Pregnant women in third trimester	FP services: FP balanced counseling strategy (no further details provided)<br><br>MCH services: Maternal child health book<br><br>Integration point: Integrated MCH and FP services	Maternal child health book as a means of IEC tool	The intervention group showed an increase in on attitudes, subjective norms, and intentions on modern contraception use
Pearson 2020, Tanzania[100]	Stepped-wedge clustered randomized controlled trial	Pre- and post-intervention assessment	Postpartum women	FP services: (1) workshops on FP and PPIUD for doctors, midwives, nurses, and general hospital staff who worked in maternity wards; (2) training of maternity care providers in hospitals and in s Ministry of Health on PPFP counseling; (3) training of doctors in study hospitals in PPIUD insertion; (4) provision of PPFP leaflets during counseling; (5) provision of a video	No intervention during waiting period	The intervention increased PPIUD counseling by 19.8 percentage points (95% CI: 9.1, 22.6) and choice of PPIUD by 6.3 percentage points (95% CI: 2.3, 8.0)<br><br>Among women counseled, determinants of choosing PPIUD included receiving an informational

				in the hospital waiting area; (6) provision of Kelley's forceps for vaginal PPIUD insertion and of copper-T IUDs to hospitals; and (7) monitoring and evaluation of counseling activities and PPIUD insertions<br><br>MCH services: Hospital staff provided routine MCH services<br><br>Integration point: PPIUD training to hospital staff		leaflet during counseling and being counseled after admission for delivery services
Pence 2007, Ghana[101]	Experimental evaluation study	Navrongo Demographic Surveillance System Data	Women aged 15–49 years	FP services: (1) Community mobilization and male involvement by community health volunteers; and (2) doorstep family planning services offered by community health nurses using motorbikes<br><br>MCH services: Routine MCH services through community nurses and volunteers in community clinics<br><br>Integration point: Provision of integrated routine MCH and FP services	Regular MCH services at the health facilities	In areas with village-based community nurse services, under-five child mortality fell by 14% during five years of program implementation compared with before the intervention, with reductions in infant (5%), early child (18%), and late child (39%) mortality
Phillips 2006, Ghana[102]	Experimental evaluation study	Navrongo Demographic Surveillance System Data	Women aged 15–49 years	FP services: (1) Community mobilization and male involvement by community health volunteers; and (2) doorstep family planning services offered by community health nurses using motorbikes<br><br>MCH services: Routine MCH services through community nurses and volunteers in community clinics<br><br>Integration point: FP services integrated into regular MCH services at the health facilities.	Regular MCH services at the health facilities	Assigning nurses to community locations reduced childhood mortality rates by over half in 3 years<br><br>Fertility reduced by 15%, representing a decline of one birth in the total fertility rate
Pleah 2016, Benin, Chad, Coˆte d'Ivoire,	Process evaluation	Service data	Pregnant, early labor and postpartum women	FP services: Three components of PPFP/PPIUD services including (1) provider training, (2) supportive supervision	None	From 2014 to 2015, more than 15,000 women were counseled about PPFP, and 2,269 women chose and received

Niger, Senegal, & Togo[103]				for trained staff, and (3) ongoing monitoring of outputs and outcomes<br><br>MCH services: Routine postpartum services; further details not provided<br><br>Integration point: FP services integrated into MCH service package		the PPIUD in Benin, Coˆte d'Ivoire, Niger, Senegal, and Togo<br><br>Two of the selected countries (Benin and Niger) have expanded their PPFP/PPUID training programs to additional sites
Pradhan 2019, Nepal[104]	Stepped-wedge clustered randomized controlled trial	Interview while in postnatal ward after delivery and before discharge from hospital	Postpartum women	FP services: (1) workshops on FP and PPIUD for doctors, midwives, nurses, and general hospital staff who worked in maternity wards; (2) training of maternity care providers in hospitals and in s Ministry of Health on PPFP counseling; (3) training of doctors in study hospitals in PPIUD insertion; (4) provision of PPFP leaflets during counseling; (5) provision of a video in the hospital waiting area; (6) provision of Kelley's forceps for vaginal PPIUD insertion and of copper-T IUDs to hospitals; and (7) monitoring and evaluation of counseling activities and PPIUD insertions<br><br>MCH services: Hospital staff provided routine MCH services<br><br>Integration point: PPIUD training to hospital staff	No intervention during waiting period	Being exposed to the intervention increased PPIUD counseling among women by 25 percentage points (95% CI: 14, 40), and PPIUD uptake by four percentage points (95% CI: 3, 6)
Puri 2020, Nepal[105]	Stepped-wedge clustered randomized controlled trial (Qualitative study)	Interviews with pregnant women	Postpartum women	FP services: (1) workshops on FP and PPIUD for doctors, midwives, nurses, and general hospital staff who worked in maternity wards; (2) training of maternity care providers in hospitals and in s Ministry of Health on PPFP counseling; (3) training of doctors in study hospitals in PPIUD insertion; (4) provision of PPFP leaflets during counseling; (5) provision of a video in the hospital waiting area; (6) provision of Kelley's forceps for vaginal PPIUD insertion and of copper-T IUDs to hospitals;	No intervention during waiting period	Overall, the quality of FP counselling during ANC was unsatisfactory based on patient expectations and experience of interactions with providers, as well as FP methods offered<br><br>Despite their interest, most women reported that they did not receive thorough information about FP, and about a third of them said that they did not receive any counselling services on PPFP

				and (7) monitoring and evaluation of counseling activities and PPIUD insertions<br><br>MCH services: Hospital staff provided routine MCH services<br><br>Integration point: PPIUD training to hospital staff		
Puri 2021, Nepal[106]	Stepped-wedge clustered randomized controlled trial	Pre- and post-intervention assessment (2-year follow-up)	Postpartum women	FP services: (1) workshops on FP and PPIUD for doctors, midwives, nurses, and general hospital staff who worked in maternity wards; (2) training of maternity care providers in hospitals and in s Ministry of Health on PPFP counseling; (3) training of doctors in study hospitals in PPIUD insertion; (4) provision of PPFP leaflets during counseling; (5) provision of a video in the hospital waiting area; (6) provision of Kelley's forceps for vaginal PPIUD insertion and of copper-T IUDs to hospitals; and (7) monitoring and evaluation of counseling activities and PPIUD insertions<br><br>MCH services: Hospital staff provided routine MCH services<br><br>Integration point: PPIUD training to hospital staff	No intervention during waiting period	Women counseled in either the pre-discharge period (Odds ratio [OR] 0.86; 95% CI: 0.80, 0.93) or in the post-discharge period (OR 0.86; 95% CI: 0.79, 0.93) were less likely to have an unmet need in the postpartum period compared to women with no counseling<br><br>Women who received counseling in both the pre- and post-discharge period were 27% less likely than women who had not received counseling to have unmet need
Routh 2001, Bangladesh[107]	Quasi-experimental study	Pre- and post-intervention population surveys and service data	Pregnant and post-partum women and their children	FP and MCH services (Strategy I): Contraceptive commodities, e.g., pills and condoms, and MCH-FP counselling services were provided to clients by female fieldworkers at locations in the community, e.g. schools and clubs, rather than in people's homes<br><br>FP and MCH services (Strategy I): A range of MCH-FP services, such as clinical and nonclinical family planning, antenatal care, postnatal care, sick childcare (including	Door-step strategy of community-based distribution of contraceptives	The clinic-based service delivery strategy was found to be a feasible alternative to the resource-intensive doorstep system in urban Dhaka. It did not adversely affect program performance and it allowed the needs of clients to be addressed holistically through a package of essential health and family planning services

				treatment of diarrhea and acute respiratory tract infections) and sick mother care were delivered from a primary health care clinic<br><br>Integration point: Delivery of MCH-FP services through alternative approaches (community vs clinic) in urban areas		
Ruhul 2001, Bangladesh[108]	Evaluation study	Household interviews (baseline and endline in intervention group and only endline in control group)	Women of reproductive age	FP services: micro-credit program for poor women with family planning components including educational campaigns and door-to-door delivery of non-clinical FP methods<br><br>MCH services: expanded program of immunization (in the first phase) and subsequently and incrementally with an essential services package in reproductive and maternal and child health (in the second phase)<br><br>Integration point: Integration of FP services with MCH services	No description provided	The contraceptive prevalence rate increased from 28.0% in 1992 to 53.0% in 1997 while the total fertility rate decreased from 4.61 in 1992 to 3.66 in 1997 in study area. The corresponding contraceptive prevalence rate and total fertility rate in the control area in 1997 were 38.41% and 4.72, respectively
Saggurti 2018, India[109]	Quasi-experimental study	Survey interview with women of self-help groups who had a live birth in the 12-months preceding survey	Women of reproductive age	FP services: Information on postpartum contraception using story cards with pictures<br><br>MCH services: Usual microcredit intervention to self-help group of women with health ªintegrationº or layering included eight weekly cycles of participatory behavior communication using different thematic modules, on maternal, neonatal, child health and promoting collectivization processes facilitated by community health facilitators<br><br>Integration point: Integrated MCH service package with FP services	Usual microcredit intervention to self-help group of women	Women from the SHGs with health intervention, relative to controls over time (time 1 to time 2), were more likely to: use contraceptive methods, have institutional delivery, practice skin-to-skin care, delay bathing for 3 or more days, initiate timely breastfeeding, exclusively breastfeed the child, and provide age-appropriate immunization
Scanteianu 2022, Rwanda[110]	Qualitative study	FGDs with FP providers and in-	Reproductive age women	FP services: CHWs served as the first point of contact for women seeking FP services—and they connect women with the next tier	None	Results indicate a well-coordinated public family planning service delivery system with community health workers

		depth interviews with FP users		of health care provider for continued care of the needs. CHWs also provided FP information to clients when providing other services as a method of FP outreach<br><br>MCH services: CHWs provided a broad range of services to mothers and their children in the community and at people's homes<br><br>Integration point: Integration of FP services MCH services		and nurses filling different and complementary roles in meeting family planning client needs at the local level
Sebastian 2012, India[111]	Evaluation study	Pre- and post-intervention survey interviews (baseline; at 4 and 9 months postpartum)	Pregnant women	FP services: Auxiliary nurse midwives (ANMs) received special training in the insertion and removal of IUD. Using communication materials, CHWs educated pregnant women and their mother-in-law on healthy timing and spacing of pregnancy, postpartum care, the lactational amenorrhea method and postpartum contraception.<br><br>MCH services: Frontline workers provided routine maternal care services as per government program<br><br>Integration point: FP services integrated into MNCH service package	Usual care (No activities other than those of the government-run health program)	At four months postpartum, women in the intervention group were more likely to know the healthy spacing messages than those in the comparison group<br><br>At nine months postpartum, women in the intervention group, those with higher knowledge of healthy spacing practices and those with correct knowledge of two or more spacing methods were more likely than others to be using a contraceptive method
Senderowicz 2023, Tanzania[112]	Stepped-wedge clustered randomized controlled trial	Interviews during pregnancy or immediately postpartum	Postpartum women	FP services: (1) Workshops on FP and PPIUD for doctors, midwives, nurses, and general hospital staff who worked in maternity wards; (2) training of maternity care providers in hospitals and in s Ministry of Health on PPFP counseling; (3) training of doctors in study hospitals in PPIUD insertion; (4) provision of PPFP leaflets during counseling; (5) provision of a video in the hospital waiting area; (6) provision of Kelley's forceps for vaginal PPIUD insertion and of copper-T IUDs to hospitals;	No intervention during waiting period	The likelihood of receiving counselling on any non-IUD method decreased among those exposed, while the likelihood of being counselled on an IUD alone was 14% points higher among the exposed (95% CI: 0.06, 0.22), suggesting this intervention increased IUD-specific counselling but reduced informed contraceptive choice

				and (7) monitoring and evaluation of counseling activities and PPIUD insertions<br><br>MCH services: Hospital staff provided routine MCH services<br><br>Integration point: PPIUD training to hospital staff		
Sheff 2019, Tanzania[113]	Clustered randomized trial	Pre- and post-intervention surveys with embedded qualitative investigation	Women of reproductive age	FP services: CHWs provided FP counseling to dispel method misconceptions, and distributed condoms and oral contraceptive pills for recurring users at the household level, to refer first time users and clients who sought other methods to the nearest health center or community dispensary, where depot- medroxyprogesterone acetate (DMPA), IUDs, and implants were available<br><br>MCH services: CHWs provided household visits and the mobilization for MCH service delivery<br><br>Integration point: FP services integrated into maternal and child health services via community health agents	No clear description provided	Doorstep provision of oral contraceptive pills and condoms was associated with a null effect on modern contraceptive uptake<br><br>Expanding geographic access without efforts to improve spousal and social support, respect preference for injectable contraceptives, and address perceived risk of side-effects offset the benefits of adopting contraceptives provided by community-based services
Singh 2016, Uganda[114]	Clustered randomized controlled trial	Baseline and 1-year follow-up data	Women of reproductive age	Intermittent training model for CHVs: 2-day initial training in a centralized location and on-going monthly training in their own villages from two full-time CHWs 2-3 hours per month for 10 months. Topics included: mapping, importance of antenatal care visits, encouraging facility-based delivery, early breastfeeding and immunization, neonatal care, family planning, among others<br><br>Integration point: CHVs trained to deliver integrated FP and MNCH services	High quality monthly training in their own villages for community health volunteers and centralized quarterly supervisory meetings	Regular training of CHVs at village level by full-time CHWs plus supportive monthly supervision improved reproductive health related activity by the CHV in the intervention villages

Tawfik 2014, Afghanistan[115]	Evaluation study	Service data and interviews with women at 3, 6, 12, and 18 months post-discharge	Postpartum women	FP services: Quality improvement teams were comprised of physicians, nurses, and midwives working in the maternity wards with involvement of selected staff working in the family planning units. These teams received training on quality improvement concepts and data collection and analysis. They were the leading implementers of the quality improvement process and for measuring its results<br><br>MCH services: Routine services in maternal clinics in health facilities<br><br>Integration point: Training of maternity ward staff on FP service quality improvement	Routine hospital care	Applying quality improvement methods helped providers recognize and overcome barriers to integration of family planning and postpartum services by testing changes they deemed feasible
Tebbets 2013, Ecuador and Nicaragua[116]	Evaluation study	Post-intervention survey, and qualitative investigation with youth and the peer providers	Youth	FP services: Youth Peer Providers under age 20 are trained to provide condoms, oral contraceptive pills, emergency contraception, injectable contraceptives<br><br>Comprehensive sexual and reproductive health information. Peers with needs beyond Youth Peer Providers' capacity are referred to health professionals offering youth-friendly services<br><br>Integration point: FP services included in comprehensive sexual and reproductive health education program for youth	None	High levels of contraceptive use among those served by the youth peer providers<br><br>High self-esteem, and stronger communication and decision-making skills among program participants
Thapa 2020, Nepal[117]	Evaluation study	Mixed methods study using knowledge assessment of service providers, coverage data, and qualitative investigation with	Pregnant and postpartum women	FP services: CHVs conducted mothers' group meetings in the communities every month, organized counseling sessions for pregnant women in the communities on FP, birth preparedness and the advantages of institutional delivery. CHVs identified danger signs among pregnant and post-partum mothers and newborns through their antenatal and postnatal home visits and	None	Intervention improved the FCHV's knowledge of PPFP and their community-based counseling

		users and providers		refer any with complications to health facilities<br><br>MCH services: Health promotion activities through mothers' group meetings in the communities, counseling sessions for pregnant women, birth preparedness and institutional delivery referrals by FCHVs<br><br>Integration point: Training of CHVs to integrate FP in the existing maternal and newborn health program		
Tom 1989, Sudan[118]	Evaluation study	Pre- and post-implementation surveys and analysis of process data	Women of reproductive age and their children	FP services: Information about contraceptives for birth spacing, distribution of oral contraceptives, and referral for other methods by trained midwives<br><br>MCH services: Nutrition education and breastfeeding, oral rehydration therapy for children with diarrhea, and vaccination for children under 5 by trained midwives<br><br>Integration point: Integrated FP-MCH services delivered by trained midwives	None	Considerable increase in contraceptive use from 10% prior the intervention to over 26% after the intervention implementation<br><br>A phased approach to training with the successive introduction if discrete interventions yielded better results than a single course of training in all interventions
Tran 2019, Burkina Faso[119]	Clustered randomized controlled trial	Follow-up of women to 12 months postintervention through exist interviews	Pregnant women	FP services: (1) Postpartum FP refresher course for providers, a counseling tool, supportive supervision, and availability of contraceptive services seven days a week (2) invitation letter to husbands or partners, appointment card, and a decision-making tool for clients and providers to enable a systematic counseling approach on FP<br><br>MCH services: Continuum of maternal and child care<br><br>Integration point: Clinic-based providers trained to provide FP counseling and commodities	Routine antenatal and postnatal care	At 12 months, modern contraceptive prevalence was 55% among women who received the package and 29% among those who received routine care in control clusters (adjusted prevalence ratio 1·79, 95% CI 1·30‒2·47)<br><br>Significant differences in modern contraceptive prevalence were also seen between intervention and control groups at 6 weeks (42% and 10%, respectively; and 6 months (59% and 24% respectively)

Tran 2020, Democratic Republic of Congo[120]	Clustered randomized controlled trial	Follow-up of women to 12 months postintervention through exist interviews	Pregnant women	FP services: (1) Postpartum FP refresher course for providers, a counseling tool, supportive supervision, and availability of contraceptive services seven days a week (2) invitation letter to husbands or partners, appointment card, and a decision-making tool for clients and providers to enable a systematic counseling approach on PPFP<br><br>MCH services: Continuum of maternal and childcare<br><br>Integration point: Clinic-based providers trained to provide FP counseling and commodities	Routine antenatal and postnatal care	At 12 months, 46% women in the intervention group and 35% in the control group were using modern contraceptives (adjusted prevalence ratio [aPR] 1.58, 95% CI 0.74, 3.38), with significant differences in the use of contraceptive implants (22% vs 6%; aPR 4.36, 95% CI 1.96, 9.70), but without difference in the use of short acting contraceptives (23% vs 28%; 0.92, 0.2, 2.98) and non-modern or inappropriate methods (7% vs 18%; 0.45, 0.13, 1.54)
Tu 2008, China[121]	Quasi-experimental study	Pre- and 28 months post-intervention follow-up survey interviews	Unmarried youth aged 15–24 years	FP services in comprehensive sexuality education: (1) educational materials dissemination, (2) instructional videos, (3) lectures by a professional educator, (4) small group activities, (5) counselling sessions, and (6) distribution of contraceptives and provision of pregnancy testing kits and contraceptives<br><br>Comprehensive sexuality education on pregnancy, masturbation, sex refusal skills, and life goals, etc.<br><br>Integration point FP topics included in comprehensive sexuality education	Standard services to engaged couples provided by FP units	Intervention associated with a significant increase in the frequency of contraceptive use among participants initiating sexual relations over the period of the intervention, as well as with significant reduction in use ever of the withdrawal method of contraception among all sexually active respondents compared with the control group during long-term follow-up period
Undie 2014, Kenya[122]	Quasi-experimental study	Pre- and post-intervention household survey interviews	Women of reproductive age	FP services: CHWs and CHEs conducted house-to-house outreach visits, community dialogue and action days. Training of healthcare providers and nurses on family planning. Topics covered: FP methods and misconceptions, religious opposition, self-diagnose underlying health issues, and available resources	No description provided	The intervention raised awareness on family planning and early pregnancy bleeding but not on post abortion care

				Post abortion care: Frontline workers trained on provided services for post abortion care<br><br>Integration point: Training of frontline workers on post abortion care and FP services		
Vance 2014, Ghana and Zambia[123]	Clustered randomized controlled trial	Exist interviews with women 9-12 months postpartum	Women and their children	Vaccinators were trained to screen women for pregnancy risk, deliver a birth spacing message and refer mothers interested in preventing pregnancy to co-located FP services during their child's immunization visit<br><br>MCH services: Routine child immunization services in health facilities<br><br>Integration point: FP services integrated into immunization clinics	Regular immunization services in the clinics	In both countries, there was no significant effect on non-condom FP method use (Zambia, P.0.56 and Ghana, P.0.86). Reported referrals to FP services did not improve nor did women's knowledge of factors related to return of fecundity
Weerasekera, 2018, Sri Lanka[124]	Stepped-wedge clustered randomized controlled trial	Face-to-Face interviews with women admitted for delivery at the intervention hospitals	Pregnant women who came to the study facilities for delivery care	FP services: (1) Workshops on FP and PPIUD for doctors, midwives, nurses, and general hospital staff who worked in maternity wards; (2) training of maternity care providers in hospitals and in s Ministry of Health on PPFP counseling; (3) training of doctors in study hospitals in PPIUD insertion; (4) provision of PPFP leaflets during counseling; (5) provision of a video in the hospital waiting area; (6) provision of Kelley's forceps for vaginal PPIUD insertion and of copper-T IUDs to hospitals; and (7) monitoring and evaluation of counseling activities and PPIUD insertions.<br><br>MCH services: Hospital staff provided routine MCH services<br><br>Integration point: PPIUD training to hospital staff	No intervention during waiting period	PPIUD as a method of contraception was successfully introduced into the 18 participating hospitals

Wendot 2018, Kenya[125]	Evaluation study	Pre- and post-intervention study with clients and semi-structured interviews with providers	Clients receiving any method of post abortion family planning	FP services: Quality management intervention aiming to increase uptake of highly effective methods of contraception in post abortion care. Three intervention components: (1) orientation training of providers in post abortion FP, (2) job-aide for the providers, and (3) enhanced supervision of and support to service providers<br><br>Post abortion care: Routine services; further details not provided<br><br>Integration point: FP services integrated in post abortion care through quality management	None	Same-day uptake of PAFP was higher at post-intervention compared to baseline, as was same-day uptake of LARC<br><br>Providers reported mixed opinions about the effectiveness of the intervention but most reported that the supervision visits helped them improve the quality of their services
Wu 2020, Nepal[126]	Evaluation study	Pre- and one-year post intervention implementation program data and individual semi-structured interviews clients, their spouses, and service providers.	Postpartum women	FP services: Patient-centered contraceptive counselling using the balanced counseling strategy<br><br>MCH services: Home-based routine maternal and childcare services aided by mobile technology<br><br>Integration point: Integrated reproductive, maternal, newborn, and child health intervention implemented by CHWs	None	Modern contraceptive use increased from 29% pre-intervention to 46% post-intervention
Zhu 2009, China[127]	Clustered randomized controlled trial	Pre- and post-intervention data	Women undergoing abortion	FP services:<br>Package A consisted of: (i) training of abortion service providers and provision of service guidelines, according to a standard training schedule (one day) and training module, (ii) provision of information for women (group education), and (iii) referral of women to existing FP services<br><br>Package B consisted of: (i) training of abortion service providers and provision of service guidelines, according to a standard training schedule (two days) and training	None	Both packages increased use of any contraceptive method, but the comprehensive approach also increased use of more effective methods. Odds ratios for consistent and correct use of condoms were 2.32 and 2.78, respectively, compared with the simple package<br><br>The rates of unwanted pregnancies and repeat abortions were somewhat reduced for both packages, with no

				module, (ii) group education, (iii) individual counselling of women, (iv) free provision of contraceptive materials, (v) male involvement, and (vi) referral of women to existing FP services<br><br>Abortion services: routine abortion services n abortion clinics<br><br>Integration point: Integration of abortion and FP services in clinics		significant statistical difference between them
Zivich 2018, Democratic Republic of Congo[128]	Evaluation study	Cross-sectional, secondary analysis of data from a cohort of women enrolled in a breastfeeding promotion trial	Postpartum women	FP services: FP counseling offered by health care provider during well-baby clinic visits. Modern contraceptive methods included intrauterine devices, injectables, implants, and contraception pills<br><br>MCH services: Well-baby clinic visits; details not provided<br><br>Integration point: FP services integrated in well-baby clinic visits for postpartum women	None	Only 14.3% were using a modern contraceptive method, despite availability at the clinic<br><br>Discussion with a nurse about family planning was associated with increased odds of using modern birth control relative to other methods

Online Supplementary File 4: Quality assessment of included studies

Author, and year	Relevance	Reliability	Validity	Applicability	Overall Score
Abdel-Tawab, 2008	Low	Low	Low	Low	Weak
Abdel-Tawab, 2011	Low	Low	Low	Low	Weak
Abdulahi, 2021	Low	Average	Average	Low	Weak
Achyut, 2016a	Average	Low	Average	Average	Moderate
Achyut, 2016b	Average	Average	Average	Average	Moderate
Adanikin, 2013	Low	Low	Low	Low	Weak
Ahmed, 2013	Low	Low	Low	Low	Weak
Ahmed, 2015	Average	Average	Low	Average	Moderate
Ajuwon, 2007	Average	Low	Low	Average	Weak
Ayiasi, 2016	Low	Low	Low	Low	Weak
Babalola, 2001	Average	Low	Low	Average	Weak
Balasumbramaniam, 2018	Low	Average	Average	Low	Weak
Bang, 2018	Low	Low	Low	Low	Weak
Baqui, 2011	High	High	High	High	Strong
Baqui, 2018	Average	Average	Average	Average	Moderate
Baynes, 2022	Low	Low	Low	Low	Weak
Benson, 2018	Low	Low	Low	Low	Weak
Bhadra, 2018	Average	Low	Low	Average	Weak
Biswas, 2017	Average	Low	Low	Average	Weak
Bolam, 1998	Average	Average	Average	Average	Moderate
Bongiovanni, 2005	Average	Low	Low	Low	Weak
Brooke, 2015	Average	Low	Low	Average	Weak
Camara, 2018	Low	Low	Low	Low	Weak
Ceylan, 2009	Low	Low	Low	Low	Weak
Charurat, 2010	Low	Low	Low	Average	Weak
Chin-Quee, 2016	High	Average	Low	Average	Moderate
Cooper, 2013	Average	Low	Low	Average	Weak
Cooper, 2014	Average	Average	Low	Average	Moderate
Cooper, 2015	Low	Low	Low	Low	Weak
Cooper, 2016	Average	Low	High	Low	Moderate
Coulibaly, 2021a	Average	High	Average	Average	Moderate
Coulibaly, 2021b	Average	High	Average	Average	Moderate
Davis, 2009	Low	Low	Low	Low	Weak
Delvaux, 2008	Average	Low	Low	Average	Weak
Dhital, 2020	Low	High	Average	Low	Moderate

Dimond-Smith, 2020	Low	Low	Low	Low	Weak
Douthwaite, 2005	Low	Low	Low	Low	Weak
Dulli, 2016,	High	Average	High	High	Strong
Erhardt-Ohren, 2020	Low	Low	Low	Low	Weak
Fatima, 2018	Low	Low	Low	Low	Weak
FHI 360, 2013	Average	Average	Low	Low	Weak
Fotso, 2015	Average	Low	Average	Average	Moderate
Gage, 2023	Average	Average	Low	Low	Weak
Gallo, 2013	Low	Low	Low	Low	Weak
Gua, 2022	Average	High	High	Average	Moderate
Hackett, 2020	Average	Low	Low	Average	Weak
Hamon, 2020	Average	Average	Low	Average	Moderate
Hamon, 2022a	High	Average	Low	Average	Moderate
Hamon, 2022b	Average	Average	Low	Average	Moderate
Harris-Fry, 2016	Average	Low	Average	Low	Weak
Harrison, 2020	Average	Low	Low	Average	Weak
Harrison, 2021	High	High	Average	High	Strong
Hemono, 2022	Low	Low	Low	Low	Weak
Hersh, 2018	Low	Low	Low	Low	Weak
Hodges, 2015	Average	Average	Average	Average	Moderate
Hoyt, 2021	Low	Low	Low	Low	Weak
Huang, 2014	Low	Low	Low	Low	Weak
Huber, 2010	Low	Low	Low	Low	Weak
Huber-Krum, 2020	High	High	High	Average	Strong
Jacinto, 2016	Average	Average	Low	High	Moderate
Jackson, 2016	Average	Average	Average	Average	Moderate
Jarvis, 2018	Low	Average	Low	Average	Weak
Karra, 2019	Average	High	High	Average	Strong
Kibel, 2022	Low	Low	Low	Low	Weak
Kimani-Murage, 2017	Average	Average	Average	Average	Moderate
Klinger, 2016	Low	Low	Low	Low	Weak
Komasawa, 2019	Average	Average	Average	Average	Moderate
Koroma, 2019	Low	Low	Low	Low	Weak
Krishnaratne, 2021	High	Low	Low	High	Moderate
Levy, 2021	Low	Low	Low	Low	Weak
Limbila, 2013	Low	Low	Low	Low	Weak
Lori, 2018	Average	Low	Low	Average	Weak
McPherson, 2010	Average	Low	Average	Average	Moderate

Mochache, 2018	Average	Low	Low	Average	Weak
More, 2017	Low	Low	Average	Average	Weak
Mwakangalu, 2018	Average	Low	Low	Average	Weak
Mwakangalu, 2020	Average	Low	Low	Average	Weak
Nelson, 2019	High	Low	Low	High	Moderate
Özçelik, 2020	Low	Low	Low	Low	Weak
Palinggi, 2021	Low	Low	Low	Low	Weak
Pearson, 2020	High	High	High	High	Strong
Pence, 2007	Average	Average	Average	Average	Moderate
Phillips, 2006	Low	Low	Low	Average	Weak
Pleah, 2016,	Low	Low	Low	High	Weak
Pradhan, 2019	Average	High	High	Average	Average
Puri, 2020	High	High	High	High	Strong
Puri, 2021	Average	High	High	Average	Moderate
Routh, 2001	Low	Low	Low	Average	Weak
Ruhul, 2001	Average	Low	Low	Average	Weak
Saggurti, 2018	Average	Average	Average	Low	Moderate
Scanteianu, 2022	Low	Low	Low	Low	Weak
Sebastian, 2012	Average	Average	Average	Average	Average
Senderowicz, 2023	High	High	High	High	Strong
Sheff, 2019	Average	Average	Average	Average	Moderate
Singh, 2016	Low	Low	Low	Low	Weak
Tawfik, 2014	Low	Low	Low	Low	Weak
Tebbets, 2013	High	Low	Low	High	Moderate
Thapa, 2020	Low	Low	Low	Low	Weak
Tom, 1989	Low	Low	Low	Low	Weak
Tran, 2019	Low	Low	Average	Average	Weak
Tran, 2020	Average	Average	High	Average	Moderate
Tu, 2008	Average	Low	Low	Average	Weak
Undie, 2014	Low	Low	Low	Low	Weak
Vance, 2014	High	Low	Average	High	Moderate
Weerasekera, 2018	Average	Low	Low	Average	Weak
Wendot, 2018	Low	Low	Low	Low	Weak
Wu, 2020	Low	Low	Low	Average	Weak
Zhu, 2009	Average	Average	Average	Average	Moderate
Zivich, 2018	Low	Low	Low	Low	Weak

Online Supplementary File 5: Effects of integrated family planning and other health related interventions in low- and middle-income countries

First author and year	Intervention group sample size	Control group sample size	Change in intervention	Change in control	Difference between change in intervention and control	Intervention effects as reported in primary studies
**Abdulahi, 2021**[22]						
Early initiation of breastfeeding	249	219				24.4 (14.5, 37.3)***
Exclusive breastfeeding	249	219				14.6 (3.77, 25.5)**
Breastfeeding Knowledge Questionnaire score	249	219				*0.15 (-0.10, 0.41)*
Iowa Infant Feeding Attitude Access Scale score	249	219				0.85 (0.77, 0.99)***
Good knowledge about breastfeeding	249	219				*5.38 (-3.59, 14.4)*
Positive attitude towards breastfeeding	249	219				*30.4 (23.4, 37.4)****
Infant length-for-age z scores	249	219				*0.05 (-0.30, 0.39)*
Infant weight-for-age z scores	249	219				0.15 (-0.11, 0.41)
Infant weight-for-length z scores	249	219				0.15 (-0.15, 0.46)
Infant mid-upper-arm-circumference in cm	249	219				0.25 (0.01, 0.49)**
Stunted	249	219				-0.25 (-1.11, 0.61)
Underweight	249	219				-0.28 (-1.38, 0.82)
Wasted	249	219				1.16 (0.00, 2.233)
**Adanikin, 2013**[25]						
Contraceptive intentions after counseling (modern method)	ANC: 101<br>PNC: 99				4.9 pp	
Contraceptive intentions after counseling (modern method)	ANC: 101<br>PNC: 99				2.3 pp	
Contraceptive intentions after counseling (undecided)	ANC: 101<br>PNC: 99				-2.6 pp	
Use of any method of contraception during postpartum	ANC: 101<br>PNC: 99				9.5 pp	
Use of traditional contraceptive method during postpartum	ANC: 101<br>PNC: 99				-12.5 pp	
Use of modern contraceptive method during postpartum	ANC: 101<br>PNC: 99				22.0 pp	

**Ahmed, 2015**[27]						
Postpartum prevalent rate of LAM at 6 months	1852	1786	9.9 pp	Not mentioned	-	
Postpartum prevalent rate of oral contraceptives at 24 months	2029	2012			5.2 pp	
Postpartum prevalent rate of condom at 24 months	2029	2012			5.6 pp	
Postpartum prevalent rate of injectables at 24 months	2029	2012			0.4 pp	
Postpartum prevalent rate of IUD/implants at 24 months	2029	2012			0 pp	
Postpartum prevalent rate of sterilization at 24 months	2029	2012			1.2 pp	
Postpartum prevalent rate of withdrawal at 24 months	2029	2012			-1.3 pp	
Postpartum prevalent rate of any method user at 24 months	2029	2012			11.4 pp	
**Ajuwon, 2007**[28]						
Mean knowledge scores of reproductive health	Intervention 1: 259<br>Intervention 2: 257<br>Intervention 3: 248	Control: 246	Intervention 1: 1.5<br>Intervention 2: 3.4<br>Intervention 3: 5	-0.2		
Discussion of reproductive health (mean)	Intervention 1: 259<br>Intervention 2: 257<br>Intervention 3: 248	Control: 246	Intervention 1: 0.5<br>Intervention 2: 0.7<br>Intervention 3: 0.9	0		
Mean scores for attitudes towards contraceptives	Intervention 1: 259<br>Intervention 2: 257<br>Intervention 3: 248	Control: 246	Intervention 1: 0.3<br>Intervention 2: 0.4<br>Intervention 3: 0.8	-0.4		

Mean scores for perceived self-efficacy for safe sex	Intervention 1: 259<br>Intervention 2: 257<br>Intervention 3: 248	Control: 246	Intervention 1: 1.1<br>Intervention 2: 0.2<br>Intervention 3: 2.3	-0.8		
Proportion that had ever had sex	Intervention 1: 259<br>Intervention 2: 257<br>Intervention 3: 248	Control: 246	Intervention 1: 20.4 pp<br>Intervention 2: 9.6 pp<br>Intervention 3: 4.4	5.7 pp		
Proportion who used condom last sex act	Intervention 1: 259<br>Intervention 2: 257<br>Intervention 3: 248	Control: 246	Intervention 1: 19.6 pp<br>Intervention 2: 240.8 pp<br>Intervention 3: -24.2 pp	20.8 pp		
**Ayiasi, 2016**[29]						
Initiation of breastfeeding	601	731				1.16 (0.51, 2.61)
Offer of pre-lacteal feeds	601	731				1.09 (0.56, 2.03)
Current contraceptive users	580	733				1.10 (0.51, 2.40)
Willig to use contraceptive	298	497				0.98 (0.53, 1.82)
Pregnancy test	627	758				0.50 (0.13, 1.87)
**Balasumbramaniam, 2018**[31]						
Use of family planning	1220	824	19.1 pp***	1.1 pp		
DPT immunization given to child	1220	824	7.8 pp**	9.9 pp***		
**Bang, 2018**[32]						
Use of contraceptive method			30.5 pp	2.5 pp		
Use of antenatal care			8.5 pp	-5.5 pp		
Institutional childbirth			82.7 pp	62.5 pp		
Use of postpartum checkup			26.0 pp	-38.0 pp		
**Baqui, 2011**[33]						
Use of any contraceptive method at 36 months postpartum	2117	2122	45%	39%		
Use of LAM at 36 months	2047	2024	1%	0%		

Use of contraceptive pills at 36 months	2047	2024	15%	15%		
Use of condoms at 36 months	2047	2024	6%	4%		
Use of injectables at 36 months	2047	2024	10%	9%		
Use of IUD/implants at 36 months	2047	2024	5%	3%		
Use of sterilization at 36 months	2047	2024	6%	4%		
Use of withdrawal at 36 months	2047	2024	2%	3%		
Any method users at 36 months	2047	2024	45%	39%		
**Baqui, 2018**[34]						
Risk of short birth interval	2168	2156				0.81 (0.69, 0.95)**
Risk of preterm birth	2168	2156				0.79, 0.63, 0.99)**
**Bolam, 1998**[39]						
Exclusive breast feeding	204	199				1.00 (0.67, 1.49)
Appropriate immunization	205	198				1.18 (0.67, 2.08)
Any contraceptive used for family planning	203	199				1.49 (0.87, 2.53)
**Camara, 2018**[42]						
Use of any FP method	Routine counseling: 187<br>Reinforced counseling: 194		Routine counseling: 4.8%<br>Reinforced counseling: 5.7%			
Use of a modern FP method	Routine counseling: 187<br>Reinforced counseling: 194		Routine counseling: 3.2%<br>Reinforced counseling: 4.6%			
**Cooper, 2016**[49]						
Knowledge of optimal birth spacing			14.0	3.8		1.68***
Does not want another pregnancy in next two years			-1.2	-3.3		1.12
Joint contraceptive decision making			16.3	5.5		2.19
At risk of pregnancy			0.1	8.7		0.67***
Use of modern method			-5.6	-14.8		1.45***

Use of modern method by women with children ≤11 months			-7.7	-11.4		1.13
Use of modern method by women with children 12-24 months			-3.0	-18.5		1.94***
**Coulibaly, 2021a**[50]						
Use of long acting and reversible contraceptives at 12 months postpartum in Burkina Faso	284	283				2.11 (1.48, 3.02)***
Use of long acting and reversible contraceptives at 12 months postpartum in DR Congo	274	279				3.48 (2.08, 5.84)***
Use of long acting and reversible contraceptives at 12 months postpartum (pooled data)	558	562				2.49 (1.86, 3.34)***
**Coulibaly, 2021b**[51]						
Use of modern and appropriate methods at 12 months postpartum	238	247	58.0%	47.4%		1.21 (0.91, 1.61)
Use of long-acting methods at 12 months postpartum	238	247	37.4%	27.5%		1.35 (1.08, 1.69)
Use of short-acting methods at 12 months postpartum	238	247	20.6%	19.8%		1.00 (0.55, 1.81)
Use of non-modern or non-appropriate methods at 12 months postpartum	238	247	0.4%	0%		
No contraceptive use at 12 months postpartum	238	247	42.0%	53.0%		0.8 (0.56, 1.15)
**Dulli, 2016**[57]						
Currently using a modern contraceptive	Baseline: 403<br>Endline: 426	Baseline:403<br>Endline: 422	8.0 pp	-6.8 pp		0.15 (0.01, 0.29)
Unmet need for contraception	Baseline: 403<br>Endline: 426	Baseline:403<br>Endline: 422	9.2 pp	6.1 pp		
Unmet need to space	Baseline: 403<br>Endline: 426	Baseline:403<br>Endline: 422	5.6 pp	-3.5 pp		
Unmet need to limit	Baseline: 403<br>Endline: 426	Baseline:403<br>Endline: 422	3.6 pp	-3.6 pp		
**FHI 360, 2013**[60]						
Contraceptive use			8.0 pp	-7.0 pp	15.0 pp**	
**Gage, 2023**[62]						
Currently using implant	415	346				0.238 (0.159, 0.317)***
Currently using injectable	415	346				-0.060 (-1.21, 0.002)
**Guo, 2022**[64]						
Incident pregnancy						**-0.007 (-0.030, 0.014)**

**Harrison, 2020**[70]						
Using a method by 3 months	108	100				1.3 (1.1, 1.5)***
Using a short acting method by 3 months	108	100				0.98 (0.8, 1.1)
Using the implant by 3 months	108	100				1.3 (1.2, 1.4)***
**Harrison, 2021**[71]						
Implant uptake	108	100	25%	3%		
**Hersh, 2018**[73]						
Mean family planning knowledge score (possible total score 7)	Conversation group: 119<br>Video group: 121				Conversation group: 2<br>Video group: 2	
**Huber-Krum, 2020**[78]						
Modern contraception at year-1 follow-up	19298				4.4 pp	0.04 (-0.0, 0.10)
Short-acting contraception at year-1 follow-up	19298				-3.0 pp	0.02 (-0.02, 0.07)
Long-acting reversible contraception at year-1 follow-up	19298				7.4 pp	0.03 (0.01, 0.05)**
Sterilization at year-1 follow up					-0.3	-0.01 (-0.02, -0.00)**
Modern contraception at year-2 follow-up	19248				1.9 pp	0.00 (-0.04, 0.04)
Short-acting contraception at year-2 follow-up	19248				-5.0 pp	-0.01 (-0.04, 0.02)
Long-acting reversible contraception at year-2 follow-up	19248				6.9 pp	0.02 (-0.00, 0.04)
Sterilization at year-2 follow up					0 pp	-0.01 (-0.02, 0.00)
**Karra, 2019**[82]						
Choice of PPIUD						0.027 (0.000, 0.054)
**Kimani-Murage, 2017**[84]						
Exclusive breastfeeding for six months	420	458	55.2%	54.6%		1.11 (0.61, 2.02)
**Komasawa, 2019**[86]						
Modern contraceptive use	Baseline 434<br>Endline 426	Baseline 432<br>Endline 435	4.9 pp	0.6 pp	4.3 pp	
Traditional contraceptive use	Baseline 434<br>Endline 426	Baseline 432<br>Endline 435	1.7 pp	2.2 pp	-0.5 pp	
Spousal agreement on contraception	Baseline 434<br>Endline 426	Baseline 432<br>Endline 435	6.0 pp	0.9 pp	5.1 pp	
**More, 2017**[94]						
Use of modern contraception to space pregnancies	3439	3134				1.29 (1.06, 1.58)

Use of modern contraception to limit pregnancies	3439	3134				1.44 (1.21, 1.71)
Planned pregnancy in previous 2 years	2532	2575				1.20 (0.84, 1.73)
Malnutrition in children aged 0-59 months (severe acute)						
Malnutrition in children aged 0-59 months (moderate acute)	4608	4570				0.73 (0.48, 1.13)
	4608	4570				0.95 (0.78, 1.17)
**Palinggi, 2021**[99]						
Modern contraception	17	17	14/17	7/17		
Attitude towards contraceptive	17	17	5.4	0.7		
Subjective norms of contraception	17	17	1.6	0.07		
**Pearson, 2020**[100]						
Women's choice of PPIUD	Group 1: 8968<br>Group 2: 5982					0.063 (0.023, 0.08)
**Pradhan, 2019**[104]						
PPIUD uptake	Group 1: 6533<br>Group 2: 7366					**0.044 (0.028, 0.064)***
**Puri, 2020**[105]						
Unmet need for modern contraception	Year 1: 17799<br>Year 2: 17809		54.2%	49.6%		
Unmet need for spacing	Year 1: 17799<br>Year 2: 17809		30.5%	28.9%		
Unmet need for limiting	Year 1: 17799<br>Year 2: 17809		23.7%	20.7%		
**Routh, 2001**[107]						
Contraceptive prevalence rate	Hazaribag 400 & Gandaria 400	Siddiquebazar 400 & Wari 400	Hazaribag 4% & Gandaria 1%	Siddiquebazar 0% & Wari 1%		
**Saggurti, 2018**[109]						
Exclusive breastfeeding	374	171	17.5 pp	-8.8 pp		26.7 (9.4, 44.1)***
Use of modern methods of contraception	374	171	11.7 pp	2.6 pp		9.3 (1.3, 17.2)**
Use of modern spacing methods	374	171	7.0 pp	3.5 pp		3.3 (-0.4, 7.0)
Use of traditional methods for spacing	374	171	4.9 pp	0 pp		4.9 (1.9, 7.9)
**Sheff, 2019**[113]						

Modern contraceptive use	Baseline 508<br>Endline 393	Baseline 1032<br>Endline 859	-0.03 pp	-0.4 pp		
**Tran, 2019**[119]						
Use of modern and appropriate contraceptive methods at 12 months	286	285				1.79 (1.30, 2.47)
Use of long acting or permanent contraceptive methods at 12 months	286	285				1.66 (1.17, 2.35)
Use of short-acting contraceptive methods at 12 months	286	285				2.01 (1.18, 3.43)
**Tran, 2020**[120]						
Use of modern and appropriate contraceptive methods at 12 months	286	290				1.58 (0.74, 3.38)
Use of long acting or permanent contraceptive methods at 12 months	286	290				4.47 (2.05, 9.74)
Use of short-acting contraceptive methods at 12 months	286	290				0.92 (0.29, 2.98)
**Tu, 2008**[121]						
Contraceptive use ever	Baseline 1220<br>Endline 1157	Baseline 1007<br>Endline 1092	19.8 pp	8.8 pp		
Contraceptive use at each intercourse	Baseline 1220<br>Endline 1157	Baseline 1007<br>Endline 1092	28.2 pp	13.4 pp		
Condom use ever	Baseline 1220<br>Endline 1157	Baseline 1007<br>Endline 1092	14.4 pp	11.8 pp		
Withdrawal method use ever	Baseline 1220<br>Endline 1157	Baseline 1007<br>Endline 1092	-6.9 pp	6.0 pp		
**Undie, 2014**[122]						
Ever heard about FP methods	Baseline 378<br>Endline 421	Baseline 181<br>Endline 200	5.8 pp	-0.6 pp		
Currently using FP methods	Baseline 378<br>Endline 421	Baseline 181<br>Endline 200	6.2 pp	8.7 pp		
Currently using a modern FP method	Baseline 378<br>Endline 421	Baseline 181<br>Endline 200	6.9 pp	10.5 pp		
Reported early pregnancy bleeding experience	Baseline 378<br>Endline 421	Baseline 181<br>Endline 200	3.3 pp	-7.3 pp		
Sought post abortion care services	Baseline 378<br>Endline 421	Baseline 181<br>Endline 200	15.1 pp	20.8 pp		
**Vance, 2014**[123]						

Use of a non-condom modern method in Ghana	Pre-test 646<br>Post-test 482	Pre-test 833<br>Post-test 801	2.4 pp	0.9 pp		
Use of a non-condom modern method in Zambia	Pre-test 962<br>Post-test 1506	Pre-test 2659<br>Post-test 1092	8.1 pp	4.1 pp		
**Zhu, 2009**[127]						
Use of any contraceptive method	Package A<br>pre-intervention 555<br>post-intervention 555	Package B<br>pre-intervention 634<br>post-intervention 592	3.8 pp	2.0 pp		0.81 (0.27, 2.40)
Use of effective contraceptive methods	Package A<br>pre-intervention 555<br>post-intervention 555	Package B<br>pre-intervention 634<br>post-intervention 592	2.1 pp	6.3 pp		2.03 (1.04, 3.98)
Consistent use of condoms	Package A<br>pre-intervention 555<br>post-intervention 555	Package B<br>pre-intervention 634<br>post-intervention 592	6.7 pp	25.0 pp		2.32 (1.55, 3.46)
Correct use of condoms	Package A<br>pre-intervention 555<br>post-intervention 555	Package B<br>pre-intervention 634<br>post-intervention 592	8.7 pp	30.0 pp		2.78 (1.81, 4.26)
Consistent and correct use of condoms	Package A<br>pre-intervention 555<br>post-intervention 555	Package B<br>pre-intervention 634<br>post-intervention 592	4.4 pp	31.2 pp		5.68 (3.39, 9.53)
Regular intake of the oral contraceptives	Package A<br>pre-intervention 555<br>post-intervention 555	Package B<br>pre-intervention 634<br>post-intervention 592	-20.5 pp	-12.7 pp		0.19 (0.03, 1.38)
